# Transcriptome Analysis of *Salicornia europaea* under Saline Conditions Revealed the Adaptive Primary Metabolic Pathways as Early Events to Facilitate Salt Adaptation

**DOI:** 10.1371/journal.pone.0080595

**Published:** 2013-11-12

**Authors:** Pengxiang Fan, Lingling Nie, Ping Jiang, Juanjuan Feng, Sulian Lv, Xianyang Chen, Hexigeduleng Bao, Jie Guo, Fang Tai, Jinhui Wang, Weitao Jia, Yinxin Li

**Affiliations:** Key Laboratory of Plant Molecular Physiology, Institute of Botany, Chinese Academy of Sciences, Beijing, P.R. China; East Carolina University, United States of America

## Abstract

**Background:**

Halophytes such as *Salicornia europaea* have evolved to exhibit unique mechanisms controlled by complex networks and regulated by numerous genes and interactions to adapt to habitats with high salinity. However, these mechanisms remain unknown.

**Methods:**

To investigate the mechanism by which halophytes tolerate salt based on changes in the whole transcriptome, we performed transcriptome sequencing and functional annotation by database search. Using the unigene database, we conducted digital gene expression analysis of *S. europaea* at various time points after these materials were treated with NaCl. We also quantified ion uptakes. Gene functional enrichment analysis was performed to determine the important pathways involved in this process.

**Results:**

A total of 57,151 unigenes with lengths of >300 bp were assembled, in which 57.5% of these unigenes were functionally annotated. Differentially expressed genes indicated that cell wall metabolism and lignin biosynthetic pathways were significantly enriched in *S. europaea* to promote the development of the xylem under saline conditions. This result is consistent with the increase in sodium uptake as ions pass through the xylem. Given that PSII efficiency remained unaltered, salt treatment activated the expression of electron transfer-related genes encoded by the chloroplast chromosome. Chlorophyll biosynthesis was also inhibited, indicating the energy-efficient state of the electron transfer system of *S. europaea*.

**Conclusions:**

The key function of adjusting important primary metabolic pathways in salt adaption was identified by analyzing the changes in the transcriptome of *S. europaea*. These pathways could involve unique salt tolerance mechanisms in halophytes. This study also provided information as the basis of future investigations on salt response genes in *S. europaea*. Ample gene resources were also provided to improve the genes responsible for the salt tolerance ability of crops.

## Introduction

One of the major abiotic stresses that threaten agriculture production is saline soil, which account for approximately 20% of the world’s cultivated lands [1-3]. Considerable efforts have been devoted to breed crops that can be harvested from saline marginal land and to determine the mechanisms of plant salt tolerance. Studies have focused on *Arabidopsis*, a model plant with a relatively low ability to survive abiotic stress [4,5]. However, researchers also recognized the potential of plants exhibiting natural tolerance in salt-affected soils to provide novel insights into this research field [6-8]. A typical example of these plants is *Thellungiella halophile* (salt cress), which has been well characterized for its ability to survive in environments with high salt content and low temperature as well as drought [9-11]. 

In addition to salt cress, halophytes are another kind of plant species that have adapted to and can thrive in highly saline environments [12-14]. Therefore, halophytes have exhibited a competitive advantage over other plants and become an important economical non-crop species that can be used for saline land reclamation; these halophytes can also be used for biofuel precursor production on large marginal lands [1,3]. Salt adaptation strategies of halophytes range from variations in morphological traits to changes at the molecular level [12,14]. However, the mechanisms leading to salt tolerance in halophytes remain largely unexplored because of the lack of information on their genome or transcriptome sequences and difficulties in plant genetic manipulation. 

With the technological advancement of sequencing platforms, high-throughput DNA sequencing has been developed to help sequence and assemble complex dynamic transcriptomes efficiently [15,16]. Furthermore, a tag sequencing method implemented on the Illumina platform, which is an analog of the serial analysis of gene expression (SAGE), has allowed the profiling in a digital format of large amounts of transcriptomes for parallel experiment materials; this method is more accurate and precise than conventional microarrays [17,18]. For the non-model plant without a reference genome, high-throughput gene expression changes can also be determined comprehensively using the tag sequencing method coupled with *de novo* transcriptome assembly [19,20]. 

As a euhalophyte without a gland or a bladder used for salt excretion, *Salicornia europaea* is a good candidate to study the inner mechanisms of halophytes coping with NaCl at the molecular level [21,22]. The mechanisms of salt tolerance of *S. europaea* have been determined by investigating the changes in proteomic networks under saline conditions and by determining several genes related to salt tolerance [23,24]. Previous studies further showed that *S. europaea* can accumulate high amounts of salt in the shoots under saline conditions [25,26]. Salt in soil can be assimilated against water potential from the roots to the aerial parts of the plant. This process requires the roots and shoots to coordinate, but this coordination is an uncharacterized process of *S. europaea*. The major altered pathways in either roots or shoots of *S. europaea* and in other halophytes when coping with salinity often indicated by differentially expressed genes have rarely been identified. Recently, the transcriptome of *S. europaea* shoot was analyzed for both salt-treated and salt-free conditions which is useful for future study of this plant [27]. However, only shoot was selected in their research, which is insufficient for profiling the whole transcriptome change in the plant. Furthermore, it will be better to understand the salt tolerance mechanism by investigating the time series change of transcriptome after salt treatment. 

In our research, the mechanism by which *S. europaea* tolerates salt on the basis of the changes in the whole transcriptome was investigated by transcriptome sequencing and digital gene expression analysis for both roots and shoots of *S. europaea* under different time of treatment. Conducting gene functional enrichment analysis, we found that the genes involved in cell wall metabolism pathways were differentially expressed to promote xylem development in *S. europaea* under saline conditions, which may facilitate salt ion uptake from the root vascular system to the shoot. Function analysis of the shoot-enriched differentially expressed genes (DEGs) showed that salt treatment particularly inhibited the genes involved in chlorophyll biosynthesis as the expression of genes involved in electron transfer and encoded by chloroplast chromosome was activated. These results indicated the energy-efficient state of the electron transfer system to generate the proton gradient and produce ATP of *S. europaea* under saline conditions. Gene Ontology (GO) comparison between *S. europaea* and *T. salsuginea* indicated that *S. europaea* contained more genes that were available in primary metabolic pathways. This result is consistent with our observation on the importance of adjusting several metabolic pathways in *S. europaea* under saline conditions. These results provided new insights into certain crucial metabolic pathways that may function in *S. europaea* to cope with salinity. This study also provided a comprehensive list of DEGs showing potential regulatory mechanisms under saline conditions.

## Materials and Methods

### Plant growth and treatment

The seeds of *S. europaea* were provided by Dafeng Jinglong Marine Industrialized Development Corporation, Ltd., Jiangsu Province, where the seeds were collected from their own field in a coastal area. Because it is their own territory, no specific permissions were required. Collecting seeds of *S. europaea* in that area did not involve endangered or protected species. The seeds were sown on vermiculite damped with tap water. After germination, the seedlings were grown in a greenhouse in Beijing Botanical Garden maintained at a thermo period of 25 °C /20 °C day/night temperature, photoperiod of 16 h, and a relative humidity of 50 ± 10%. The seedlings were irrigated weekly with half-strength Hoagland nutrient solution. After 30 d, the plants were divided into seven groups and then salinized with 200 mM NaCl for 3, 12, and 24 h and 3, 7, and 21 d. The plant group without NaCl treatment was used as the control group, which was designated as treated with NaCl for 0 h. The plant materials were treated with NaCl at different time points but were harvested at the same time to ensure that they exhibited the same biological age and growth rhythm (Figure S1 in File S1).

### RNA Isolation, cDNA Library Preparation, and RNA Sequencing

RNA was isolated from the shoot and root tissues of *S. europaea* as described previously and treated with RNase-free DNase I [28]. RNA quality was determined by agarose electrophoresis. Agilent 2100 bioanalyzer was also used to determine RNA quality. For RNA-seq analysis, equal amounts of RNA isolated from the samples treated at different times (0, 3, 12, and 24 h; and 3, 7, and 21 d) were mixed to prepare the cDNA library at the Beijing Genomics Institute. In brief, mRNAs were purified using poly-T oligo-attached magnetic beads and fragmented into 100 bp to 400 bp. The first- and second-strand cDNAs were synthesized, end repaired, and connected to the sequencing adapters. After agarose gel electrophoresis was performed, suitable fragments were enriched by PCR amplification. The library was sequenced using Illumina HiSeq™ 2000 Sequencing Systems. The raw data was uploaded into the NCBI SRA database with the accession number SRX302090.

### Transcriptome de novo assembly

After sequencing, the adaptor fragments or low-quality sequences were removed from the raw reads before data analysis. *De novo* assembly of the transcriptome sequence was performed using the short reads assembling program SOAPdenovo [29] (version 1.04; http://soap.genomics.org.cn/ soapdenovo.html). The reads were combined with a certain length of overlaps to form longer fragments without a gap called contigs. The contigs from the same transcript were then identified by paired-end reads that were connected to scaffolds. The paired-end reads were reused to fill the gap in the scaffolds and obtain sequences with the least gaps that cannot be extended on either of the ends. These sequences are defined as unigenes. To determine the sequence direction of the unigene, we performed BLASTX alignment (e-value < 0.00001) between unigenes and protein databases such as the NCBI non-redundant (Nr) protein database, Swissprot protein database, Kyoto Encyclopedia of Genes and Genomes (KEGG) pathway database, and Cluster of Orthologous Groups (COG) database. The best aligning results with priority order of Nr, Swissprot, KEGG, and COG were used to determine the sequence direction of unigenes. For the unigenes that cannot be aligned to the aforementioned databases, ESTScan [30] was introduced to predict the coding regions and sequence direction.

### Annotation and classification of unigenes

To assign gene descriptions, we searched the unigene sequences from the protein databases, including Nr, Swissprot, KEGG, and COG (e-value < 0.00001) by using BLASTX. We also retrieved the proteins with the highest sequence similarity to the given unigenes along with their protein functional annotations. The Blast2GO program [31] was utilized to obtain the GO annotations of the unigenes. WEGO software was used to perform GO functional classification for the unigenes and understand the GO distribution of the gene functions of the species from the macro level [32]. The unigenes were further aligned to COG, KEGG, and MapMan databases to predict and classify the possible pathways of these unigenes.

### Digital gene expression (DGE) tag sequencing

After the tag number and sequence information were obtained, the expression level of the corresponding gene could be determined digitally similar to the LongSAGE approach [33]. Tag sequencing preparation was performed as described in a previous study [17]. In brief, 6 µg of RNA isolated from each sample was incubated with oligo-dT magnetic beads to purify mRNA and then used as a template to synthesize the first- and second-strand cDNAs. Bead-bound cDNA was subsequently digested with restriction enzyme Nla III, which recognized and cut off the CATG sites. The Illumina adaptor 1 containing a Mme I restriction site was bound to the sticky 5ʹ end of the digested bead-bound cDNA fragments. The junction of adaptor 1 and CATG site was the recognition site of the restriction enzyme Mme I, which was used to cut 21 bp downstream from that site, thereby creating a unique tag 21 bp long. After digestion, this unique tag with adaptor was ligated to adaptor 2. The tags with different adaptors at both ends were acquired to form a tag library and sequenced using the sequencing by synthesis (SBS) method on the Illumina sequencing platform. Raw sequences were transformed into clean tags after the 3ʹ adaptor fragments and a few low-quality sequences were removed.

### Characterization of DGE tag libraries

During map annotation, all of the clean tags were mapped to our *S. europaea* transcriptome reference database assembled previously with only a mismatch of 1 bp allowed. The clean tags that matched the multiple genes from the reference sequences were filtered. The other clean tags were designed as unambiguous clean tags. For the digital gene expression analysis, the number of unambiguous clean tags for each gene was calculated and normalized to TPM (the number of tag transcripts per million clean tags).

The library of the tags for each of the samples was screened for the differentially expressed tags to compare with the large-scale changes in gene expression levels in NaCl-treated plants at different time points. A statistical comparison was performed using a rigorous algorithm described in a previous study [34]. We used the false discovery rate (FDR) to determine the threshold of P value in multiple tests and analyses. FDR ≤ 0.001 and the absolute value of log2Ratio ≥ 1 were set as the threshold to determine the significance of gene expression in our experiments.

### Clustering analysis for differentially expressed genes (DEGs)

The k-means clustering of the DEGs in NaCl-treated *S. europaea* at different times were conducted for the roots and shoots with the help of GenePattern platform (Reich et al., 2006). The number of k-means clusters was independently validated using the figure of merit (FOM), which can be used to determine the optimum input parameters for a clustering algorithm. This number was also computed using MeV [35] with varying settings. Hierarchical clustering analysis was also conducted using the GenePattern platform.

### Significant enrichment analysis for DEGs based on MapMan classification

The enrichment analysis of DEGs was based on MapMan [36-38], which has several classification levels for a specific gene. Custom R scripts were used to count and determine the significantly enriched categories. In brief, all of the DEGs were mapped to different units of MapMan Bin and the number of DEGs in each of the unit was calculated. Fisher’s exact test was used to identify the MapMan Bin overrepresented in our data set when compared with the entire transcriptome background. The P value was converted to -log_10_, which can be demonstrated by Heat map viewer embedded in GenePattern platform [39]. Darker colors in the MapMan Bin represented higher degrees of functional enrichment.

### Semi-thin section observation of *S. europaea* shoots

The middle sections of the third shoot segment from the bottom were used in morphological analysis. The shoot section with a length of 0.5 cm was fixed for 1 h with 3% glutaraldehyde in 100 mM phosphate-buffered saline (PBS) under vacuum to ensure that the fixatives were filtered through the tissues. The sample was further fixed in fresh fixatives for 12 h. The samples were then washed five times with PBS buffer, dehydrated in a graded ethanol series and acetone, and embedded in resin. The semi-thin sections of the sample were cut transversely by using an ultramicrotome (Lecia) and stained with 0.5% (w/v) aniline blue. The sections were examined under a light microscope and photographed with a CCD camera. For the semi-thin section assay of different plant samples, three to five shoot fragments for each sample were used and the representative section photos were shown. 

### Quantification of sodium content in plant shoots and roots

Plant shoots and roots were washed with distilled water immediately after these samples were harvested and dried at 60 °C for 72 h in an oven. The dried tissues were subsequently ground into fine powder with a mortar and pestle. Tissue powders (300 mL) were mixed with 10 mL of 500 mM HNO_3_ and incubated at 80 °C for 1 h. After the extracts were filtered, Na element concentrations in the plant tissues were determined using inductively coupled plasma atomic emission spectroscopy (ICP-AES). 

### X-ray microanalysis

X-ray microanalysis coupled with scanning electron microscopy (SEM) was performed as described to determine the diffusible sodium element *in situ* of *S. europaea* shoots and roots [25,40]. The shoot and root samples treated at different times were washed three times with distilled water. The middle sections of the third shoot segment from the bottom were collected as the shoot sample. The sections obtained at a depth of 1.5 cm to 2 cm from the taproots were selected as the root sample. The shoot and root samples were dipped in 5% agar in a copper holder. The samples were immediately sliced by freehand using a razor blade to obtain the transverse sections and then frozen in liquid nitrogen. The samples were freeze dried in vacuum, coated with carbon in a vacuum sputter, and analyzed using an X-650 scanning electron microscope equipped with an energy dispersive X-ray detector (EDX-9100, Hitachi, Japan). Data collection and data transformation were performed as described in a previous study [25]. The relative sodium contents of the different areas of the transverse section were obtained for each sample. Five replications were prepared for each treatment.

### *In situ* hybridization

Freshly collected root and leaf tissues were fixed in 4% formaldehyde, 5% acetic acid, and 50% alcohol overnight at 4 °C. After dehydration, the tissues were embedded in Paraplast (Sigma). The sections (8 µm) were mounted on poly-L-Lys-coated microscopic slides. The selected protein-coding regions of the unigenes were used as a template to synthesize sense and antisense digoxigenin-labeled RNA probes used for hybridization according to a detailed process described previously [41]. The primer sequences generating the probes are listed in Table S9.

### Quantitative Real-time RT-PCR (qRT-PCR)

qRT-PCR was performed as described previously [23]. a-Tubulin of *S. europaea* was used as the internal control. The relative quantification method (2^-ΔΔCt^) was used to evaluate quantitative variations between different treatments. Gene specific primers were designed according to the selected unigene sequences (Table S9).

### Statistical analysis

Statistical results are represented as means ± SD. Statistical analyses were performed by one-way ANOVA and Duncan’s multiple range tests at 5% level of significance in SPSS version 12.0. (SPSS Inc., Chicago, IL, USA).

## Results

### *De novo* assembly and function annotation of *S. europaea* transcriptome

**Figure 1:**
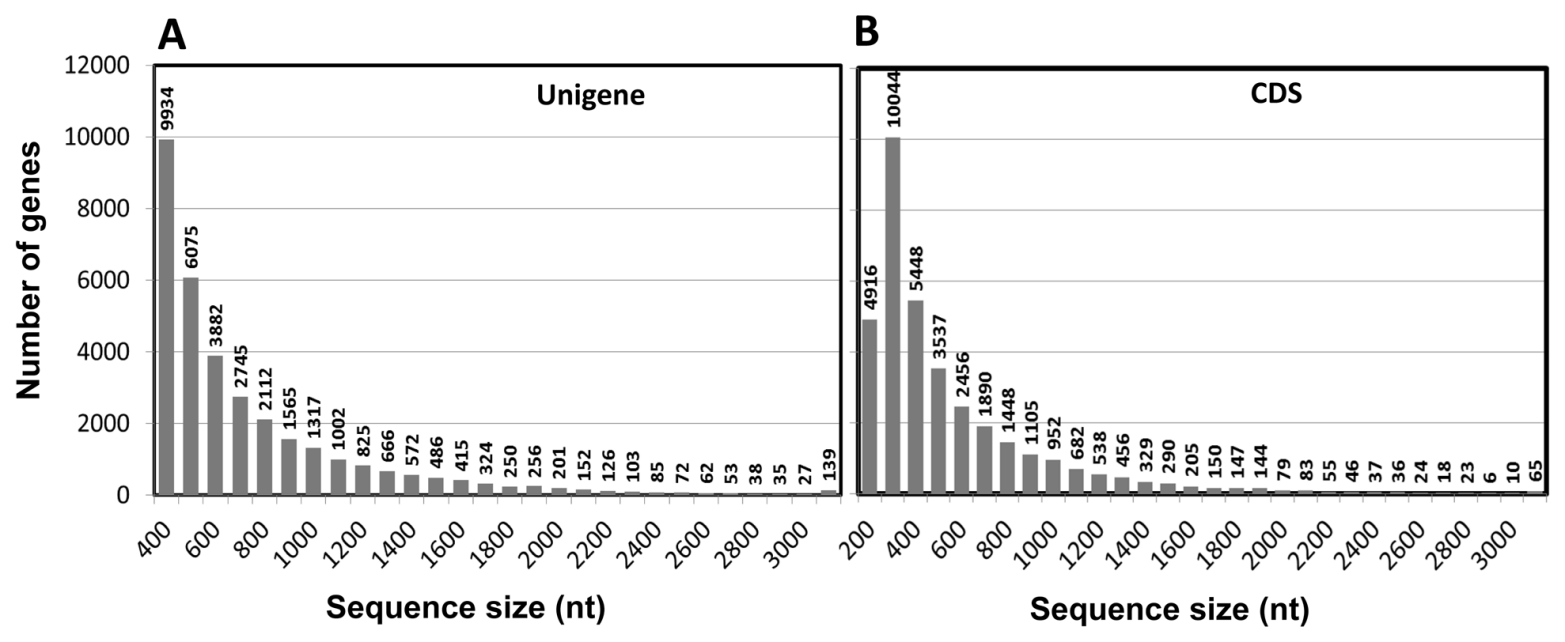
Length distribution of *S. europaea* unigenes and coding sequence (CDS) of the unigenes. (A) *S. europaea* unigenes. (B) CDS of the unigenes. The number of the unigenes and the predicted CDS were calculated within the range of preset sequence sizes.

We performed the transcriptome sequencing of *S. europaea* by using high-throughput RNA-seq technology in an Illumina sequencing platform. The output raw sequencing data were analyzed to eliminate dirty reads such as adaptors; afterward, 2000 Mbp clean reads were obtained for further analysis. Transcriptome *de novo* assembly was conducted to obtain sequences with the least gap that cannot be extended on either of the ends defined as unigenes. The length and quantity distribution of unigenes are some of the important criteria to determine the success of RNA-seq. We obtained a total of 57,151 unigenes with the length of >300 bps, in which 23,585 unigenes were >500 bps. The unigenes were used to predict the coding region sequences that were translated into amino sequences by using the standard table of codons. The results yielded 35,219 unigenes predicted with a CDS (61.62% of the total unigenes). The length distribution is shown in Figure 1.

The functional annotation of the unigenes were completed by aligning unigene sequences to the protein database Nr, Swiss-Prot, KEGG, and COG (e-value < 0.00001) by BLASTX to retrieve the proteins with the highest sequence similarity for the relevant unigene (Table S1-S4). The GO annotations of the unigenes were also obtained (Table S5). For the four protein databases, 57.13%, 38.93%, 26.12%, and 18.09% of the total unigenes were successfully annotated, respectively. MapMan is a useful tool initially designed for the model plant *Arabidopsis* [37]. MapMan comprises optimized gene classification methods designed particularly in plants that enable a better profiling of plant metabolic pathways [36,42,43]. Therefore, MapMan annotation was conducted by comparing the unigene sequences to the protein database of *Arabidopsis* and then classified 49.97% of *S. europaea* unigenes to MapMan Bin (Table S6). The genes related to proteins, RNAs, and signaling occupied the top three of the MapMan annotated unigenes were also showed (Figure S2 C in File S1). The genes that were not assigned were set aside.

### DGE Analysis of NaCl-Treated *S. europaea* at Different Times

Using the unigene database obtained by RNA-seq, we performed Taq-seq to conduct digital gene expression analysis for the roots and the aerial parts of *S. europaea* treated with 200 mM NaCl for 0, 3, 12, and 24 h and for 3 and 7 d as described (Figure S1 in File S1). The saturation analysis results indicated that the number of the detected genes almost ceased to increase when the sequencing amount reached ≥2 M, indicating sufficient coverage of the genes by clean tags (Fig. S3 A and S3 B in File S1). The distribution of clean tag expression revealed that a small number of categories of mRNA were highly expressed. By contrast, the majority of these mRNAs remained at a low level, thereby indicating the normality of the data (Figures S3C - S3F in File S1). 

All of the clean tags were characterized to judge the significant difference of gene expressions between the control sample and the other treated samples. The differentially expressed unigenes in root samples (Table S7) and shoot sample (Table S8) were obtained for further analysis. qRT-PCR was applied to verify the results from DGE analysis, with six randomly selected DEGs in shoots and roots of *S. europaea* using the designed primers (Table S9). Gene expression results from qRT-PCR were converted to log_2_ ratio as that of DEGs to compare with the same chart (Figure S4 in File S1). The correlation coefficients of the results obtained using the two counterpart methods ranged from 0.728 to 0.980, indicating that the real gene expression levels could be revealed by DGE analysis.

The presence of the assembled unigenes in *S. europaea* was further verified by RNA *in situ* analysis using the hybridization probe designed from the unigene sequence. Two representative genes, unigene 6564 and unigene 55001, were selected to characterize their distribution in the roots and shoots of *S. europaea*, respectively. Unigene 6564, which was obtained from the root longitudinal section, was annotated as an auxin transporter (PIN protein), and highly expressed in the root tips directed vertically to the base of the root cells (Figure S5 A in File S1). The location of the homologous gene in *Arabidopsis* root is similar to that in *S. europaea* and linked to the polarity of auxin transport [44,45]. Unigene 55001 is annotated as a proton-dependent oligo peptide transporter that functions in plant development and growth [46]. As shown in the slides of shoot transversal section, this gene was highly expressed in the meristematic cells of the stele cortex, a specific site showing vigorous cell growth and division (Figure S5 B in File S1). These results indicated that unigenes are present in *S. europaea*. These results are also consistent with the characteristics of the annotated gene.

**Figure 2:**
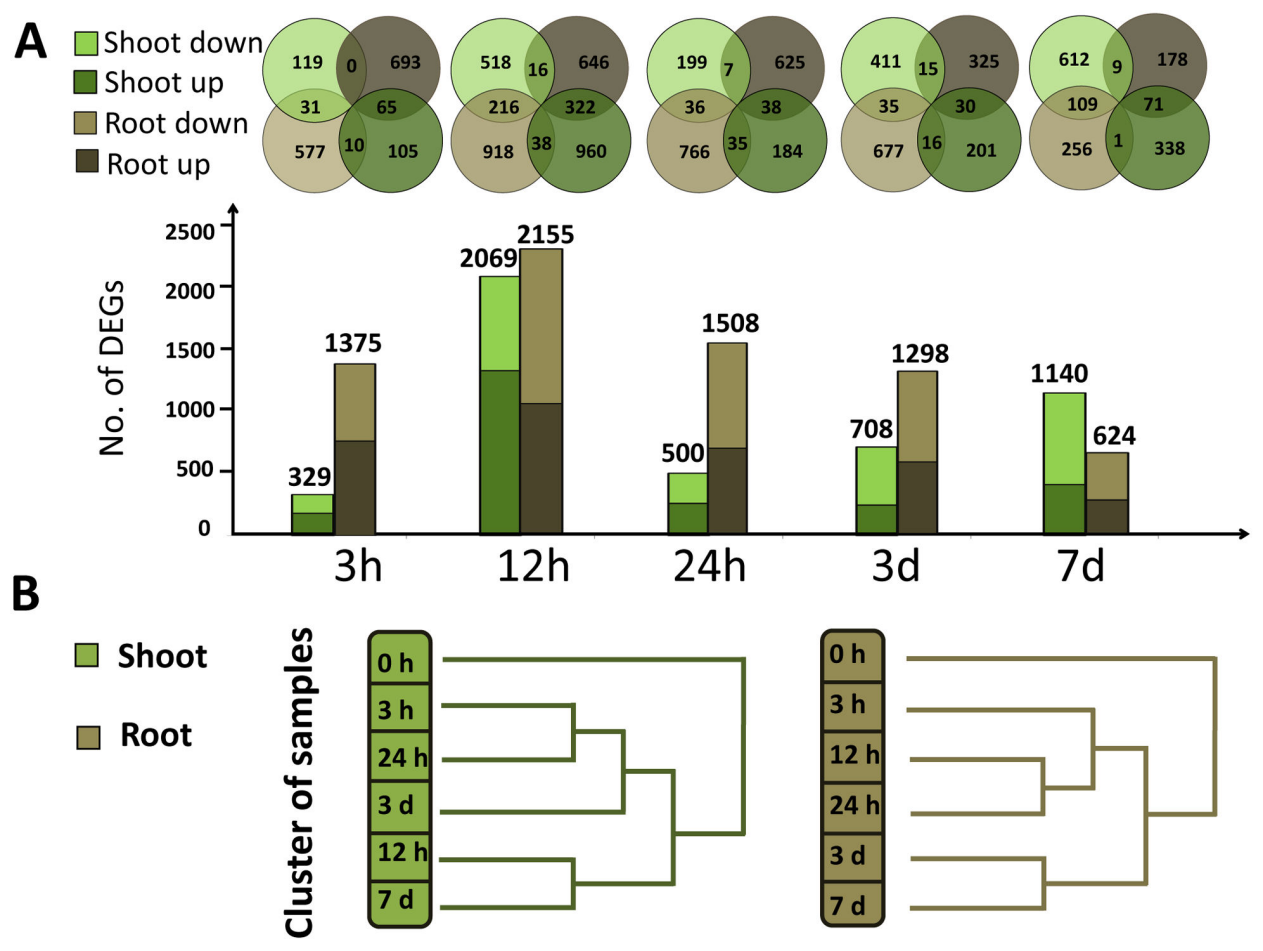
Distribution of differentially expressed genes (DEGs) of NaCl-treated plants at different time points. (A) The comparison of up-regulated and downregulated DEGs in different samples. The dark gray and light gray colors represented the up-regulated and downregulated DEGs in *S. europaea* root, respectively. The dark green and light green colors represented the up-regulated and downregulated DEGs in *S. europaea* shoot, respectively. The overlaps of the circles mean that the unigenes appeared in both of samples that the circles represent. (B) Clustering analysis of shoot and root samples treated with NaCl at different time intervals. Hierarchical clustering was conducted for the different time treated root and shoot samples based on DEGs in each sample.

 DEGs in the shoots and roots of *S. europaea* were analyzed at each time interval after treatment (Figure 2A). After 3 h of treatment, 1375 genes were differentially expressed in the roots, or the first site exposed to salinity; only 329 DEGs were identified in the shoot, which responds to environmental changes at a later time than the root. After 12 h of treatment, 2155 and 2069 DEGs were detected in the shoots and roots, respectively, which were the peak of responding genes in both tissues during the treatment period, indicating that this period was a crucial time for *S. europaea* to cope with salinity. As treatment time increased, the number of DEGs in the roots gradually decreased to 624 after 7 d, indicating that the root slowly adjusted to the saline environment. The number of DEGs in the shoot was reduced to 500 after 24 h and then increased steadily to 1140 at 7 d after the treatment (Figure 2A). The second peak of shoot DEGs may indicate that a new stage of growth in *S. europaea* shoot was initiated after the primary response to salinity. The root and shoot samples from various time points after the treatment were clustered according to the different changing patterns of DEGs. For the roots, the samples at 3 , 12, and 24 h were clustered into one group and those at 3 and 7 d were clustered into another group (Figure 2B), indicating that the DEGs may share similarities within 24 h of treatment and showed variations after a long treatment period. For shoot samples, those treated for 12 h and 7 d were clustered together and the points where the two peaks of DEGs were observed for shoot samples.

### Functional significant enrichment of DEGs reveals key response pathways to salinity in *S. europaea*

After the overall changes in DEG patterns were characterized, functional classification is necessary to determine the specific genes involved in the process. Gene functional enrichment analysis [47,48] based on MapMan Bin classification of the genes [37] can help determine the important metabolic or signaling pathways of DEGs based on plant metabolism. The significantly enriched MapMan Bin terms of DEGs were determined in *S. europaea* roots or shoots with the P value converted to -log_10_ and then demonstrated using the Heat map viewer [39].

**Figure 3:**
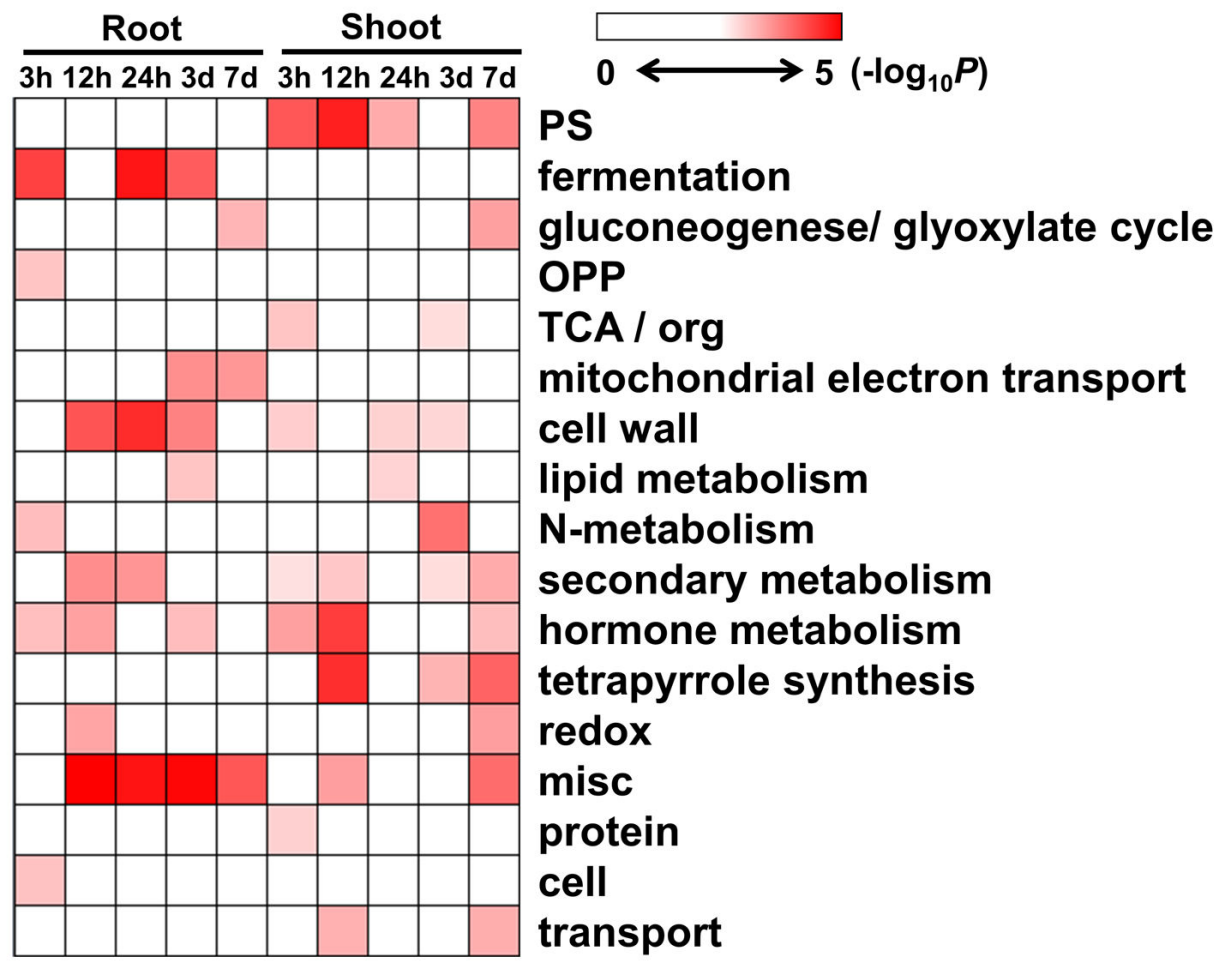
Gene significant enrichment analysis of *S. europaea* root and shoot. Fisher’s exact test was used to identify the MapMan Bin overrepresented in our dataset when compared with the whole transcriptome background. DEGs from different time intervals of NaCl treated samples were classified based on the first level of MapMan Bin classification. The P value was converted to -log_10_ and demonstrated by Heat map viewer. The darker color for the MapMan Bin represented the higher degree of the functional enrichment appeared.

Considering that MapMan Bin contains multilevel categories, we performed the functional enrichment analysis of the DEGs based on the first (Figure 3) and second levels of classification (Figure S6, S7 in File S1). At the first level, most of the enriched categories of the root DEGs were found in 12 and 24 h as well as 3 d after NaCl treatment, including the genes involved in cell wall and hormone metabolism as well as fermentation. For the shoot DEGs, the categories of photosynthesis, tetrapyrrole synthesis, and cell wall and hormone metabolism were significantly enriched at 12 h and 7 d after treatment. 

The analysis based on the second level of classification provided more details of the enriched categories (Figures S6 and S7 in File S1). The enriched categories of *S. europaea* shoots on the second level of MapMan classification were similar to those on the first level, which consisted of the genes involved in light reaction and Calvin cycle of photosynthesis. The significantly enriched categories in the shoots also included enzymes of the tetrapyrrole synthesis pathway, such as uroporphyrinogen decarboxylase and magnesium chelatase. Most of these categories showed downregulated patterns after NaCl treatment. For the roots and shoots, the significantly enriched terms consisted of genes related to cell wall metabolism, including cell wall proteins, cell wall precursor synthesis, cell wall modification, and cell wall degradation. Interestingly, most of the enriched genes involved in cell wall synthesis were downregulated, whereas the cell wall degradation genes were up-regulated. The genes associated with pectin methylesterase inhibitor family protein were also significantly enriched. 

### Dynamic changes in the transcriptome of *S. europaea* revealed by cluster analysis

**Figure 4:**
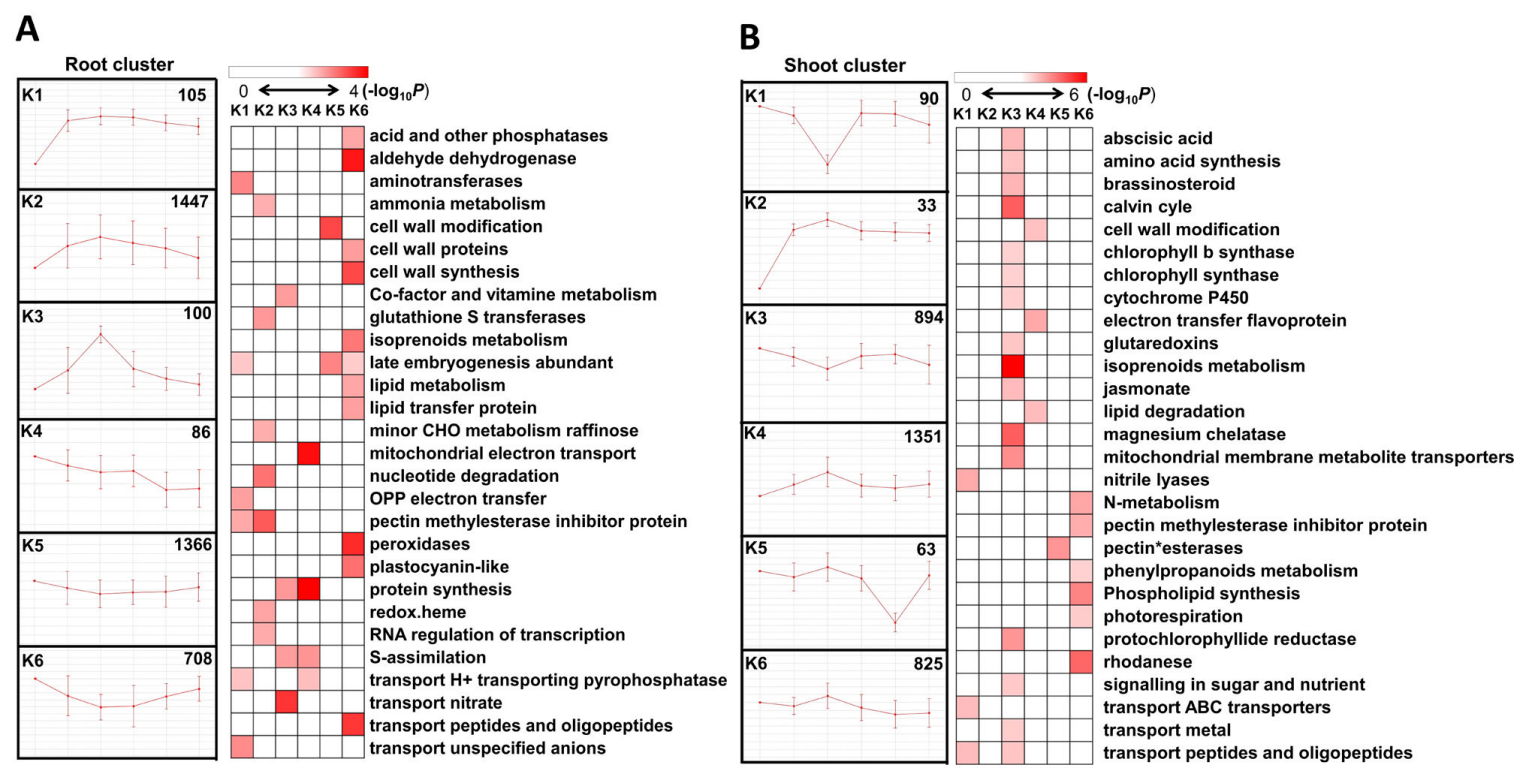
Dynamic changes in the transcriptome of *S. europaea* revealed by cluster analysis. DEGs in *S. europaea* root (A) and shoot (B) were analyzed by k-means cluster analysis followed by functional enrichment analysis for the genes in different clusters, based on the second level of MapMan Bin classification. Fisher’s exact test was used to identify the MapMan Bin overrepresented in each of the cluster when compared with the whole transcriptome background. The P value was converted to -log_10_ and demonstrated by Heat map viewer. The darker color for the MapMan Bin represented the higher degree of the functional enrichment that appeared.

To investigate the dynamically changing expression patterns of DEGs, we performed k-means clustering to study the correlated groups exhibiting similar expression profiles in *S. europaea* root (Table S10) and shoot (Table S11), where k = 6 based on an FOM calculation (Figure S8 in File S1) [35]. The enriched MapMan functional categories were tested based on the second level of classification of each cluster in *S. europaea* roots and shoots (Figure 4). K2 contained the highest amounts of DEGs of the six clusters of the root samples, and the expressions were increased in 12 h and subsequently decreased. The genes encoding the enzymes of the pectin methylesterase inhibitor family protein, glutathione S transferases, and RNA transcription were greatly enriched in k2. The enriched categories of k5 and k6 mainly included cell wall metabolism and peroxidase, in which the expressions decreased at the initial stage after NaCl treatment and gradually recovered afterward. The expression profile of k1 cluster, including the enriched categories of pectin methylesterase inhibitor family protein, aminotransferase, and unspecific anion channel was unique and showed constant upregulation after NaCl treatment. 

K4 and k6 of the shoot clusters showed slightly increasing patterns at 12 h after the treatment and contained enriched categories of the enzymes involved in cell wall modification, pectin methylesterase inhibitor family protein, and nitrogen metabolism. With the highest degree of functional enrichment, k3 accounted for approximately half of the enriched categories in the six shoot clusters, including the genes involved in Calvin cycle, isoprenoid metabolism, and tetrapyrrole synthesis-related enzymes such as chlorophyll synthase, magnesium chelatase, and protochlorophyllide reductase. 

### Morphological change and sodium element distribution of *S. europaea* after different times of NaCl treatment

 Previous studies showed that *S. europaea* can show various growth conditions after this species is treated with different NaCl concentrations for 21 d, in which 200 mM to 400 mM NaCl significantly promotes shoot growth and increased fresh weight, water content, and sodium element content of the aerial parts of the plant [24]. The present study focused on the mechanism by which the growth state and sodium element distribution of *S. europaea* changes over time under saline conditions. 

**Figure 5:**
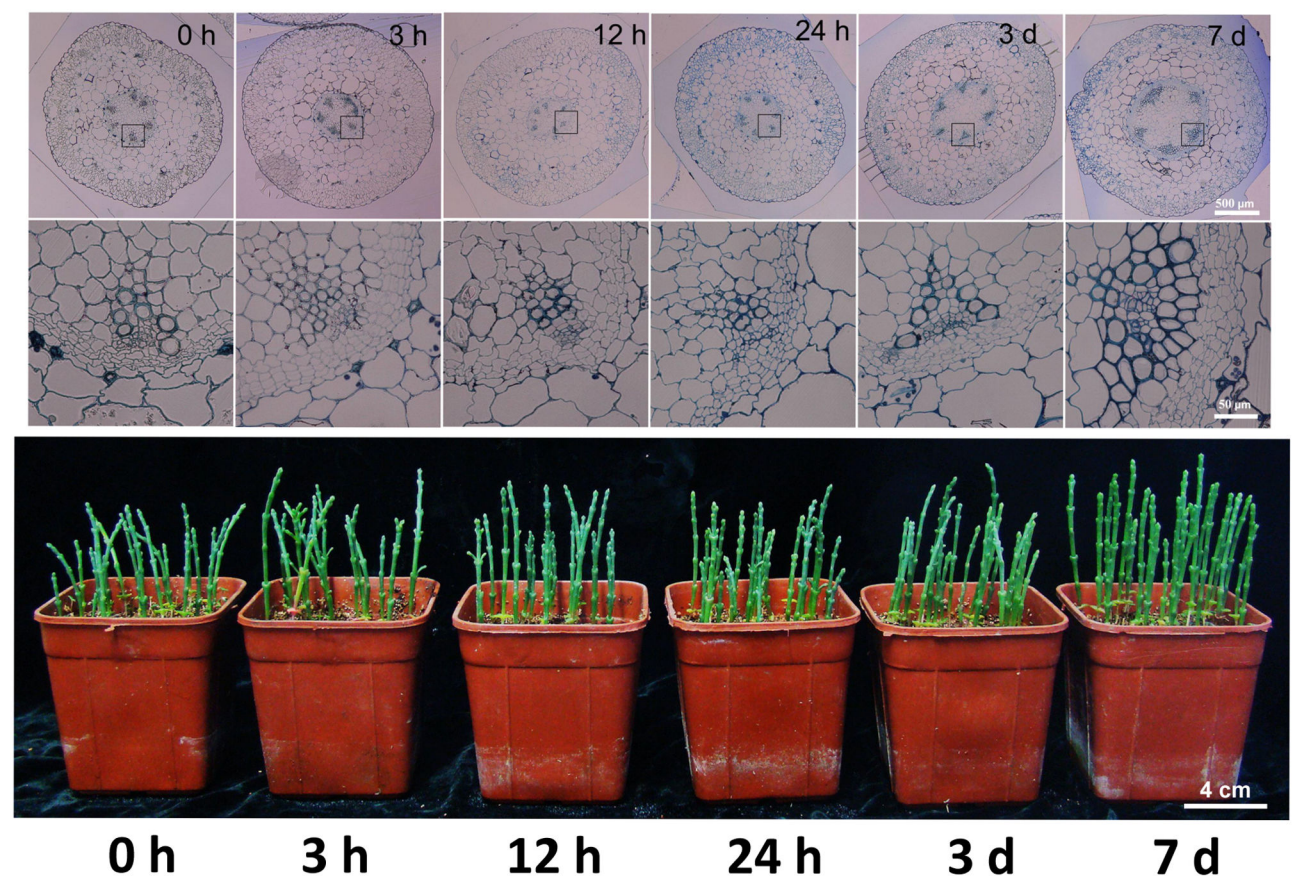
Semi-thin section observation of the *S. europaea* shoots after salt treatment. (A) The phenotype of *S. europaea* shoot after salt treatment for different time intervals. (B) The transverse section of the third internode from the base of the *S. europaea* shoot at different time intervals of NaCl treatment. The close-up image of the shoot xylem was shown underneath. The bar is equal to 500 μm.

The third node from the shoot base of *S. europaea* was selected to generate semi-thin transversal section slides and used to observe the morphological changes in *S. europaea* shoots under saline conditions with the passage of time. Figure 5 shows that the shoots remained unchanged for 24 h, but the shoot steles expanded, the xylem differentiated, and the number of xylem vessels increased at 3 d after the treatment. These results indicated that salt could promote the development of *S. europaea* xylem, which may be considered as an important factor of adaptation of this plant to salinity. 

Ion toxicity is considered as one of the a major stresses that plants under saline conditions experience and often caused by excess Na^+^ [11]. As one of the key element in plants, potassium can facilitate various metabolic reactions by activating the catalyzing enzyme. The competitive relationship between Na^+^ and K^+^ assimilation in plant has been the focus of research [49]. Therefore, the time and space distributions of sodium and potassium absorbed by *S. europaea* from the roots to the shoots after salt treatment were systematically analyzed in this study. 

**Figure 6:**
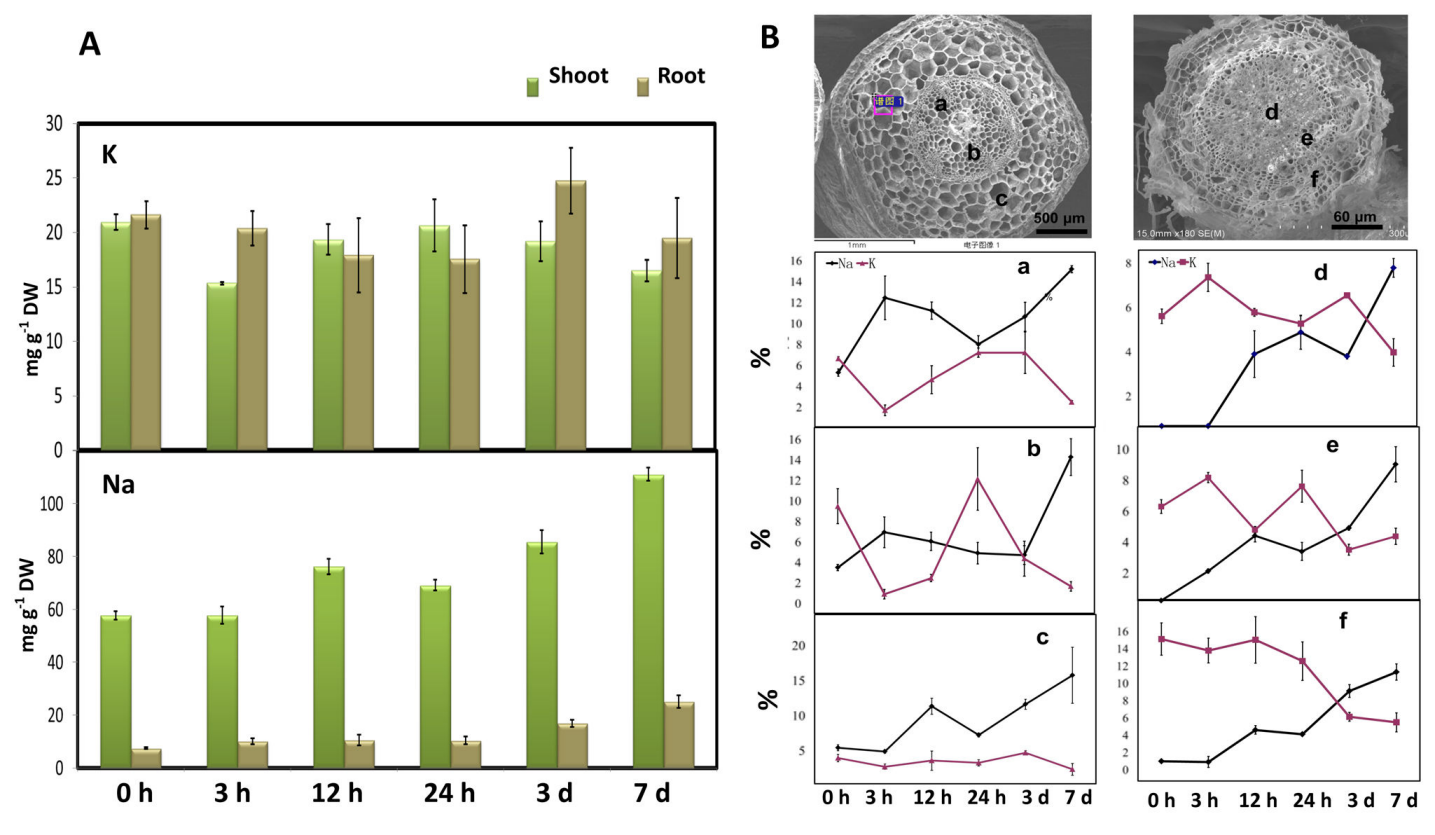
Characterization of the concentration and distribution of sodium and potassium in NaCl-treated *S. europaea* at different time points. (A) The total sodium and potassium contents in *S. europaea* shoot and root after different time intervals of NaCl treatment. (B) SEM X-ray microanalysis of sodium and potassium element distribution in *S. europaea* shoots and roots at the tissue level. The relative sodium and potassium contents were measured from different parts of the transversal sections. (a) Xylem in the shoot stele; (b) parenchyma in the shoot stele; (c) parenchyma in the shoot endodermis; (d) root medulla; (e) xylem in root stele; and (f) root exodermis.

Total potassium and sodium contents were measured by ICP-AES of *S. europaea* roots and shoots after the samples were treated at different periods (Figure 6 A). The sodium content in the roots began to increase at 3 h of treatment and remained stable for 24 h. Afterward, the sodium content increased significantly at 3 d and reached the highest content at 7 d. The sodium content in the shoots remained stable at 3 h after treatment but reached the highest level at 7 d. Potassium exhibited a different performance from sodium, in which the potassium content decreased in the roots and shoots of *S. europaea* after 7 d of NaCl treatment compared with the control group. 

X-ray microanalysis coupled with SEM can provide a sensitive technique that can be used to study *in situ* distribution of elements at the tissue level by data conversion and statistical analysis to obtain the relative percentage of element contents that have been characterized in several plants [25,26,50]. In this research, the transverse sections of *S. europaea* roots and shoots were scanned by X-ray microanalysis from the outermost to the middle tissues to determine the distribution changes in the relative potassium and sodium contents. For the shoots, we mainly focused on the stelar xylem, stelar parenchyma, and endodermis parenchyma. For the roots, the medulla, stelar xylem, and exodermis cells were considered for analysis. Figure 6B shows that the relative percentage contents of sodium and potassium from the three selected regions of the roots gradually increased with the passage of the treatment time, showing a similar trend to that of the total sodium and potassium content in the root obtained by ICP-AES. Elevated relative sodium content could be observed in the shoot stellar xylem at 3 h after NaCl treatment, which is different from the result of ICP-AES. After 12 h of treatment, the sodium content in the shoot stelar parenchyma was recorded to have significantly increased. These results indicated that sodium could be transported to the shoot through the stellar xylem within a short period after the treatment and then started to accumulate in the endodermis parenchyma. 

### Transcription factor modulation on *S. europaea* development under saline conditions

**Figure 7:**
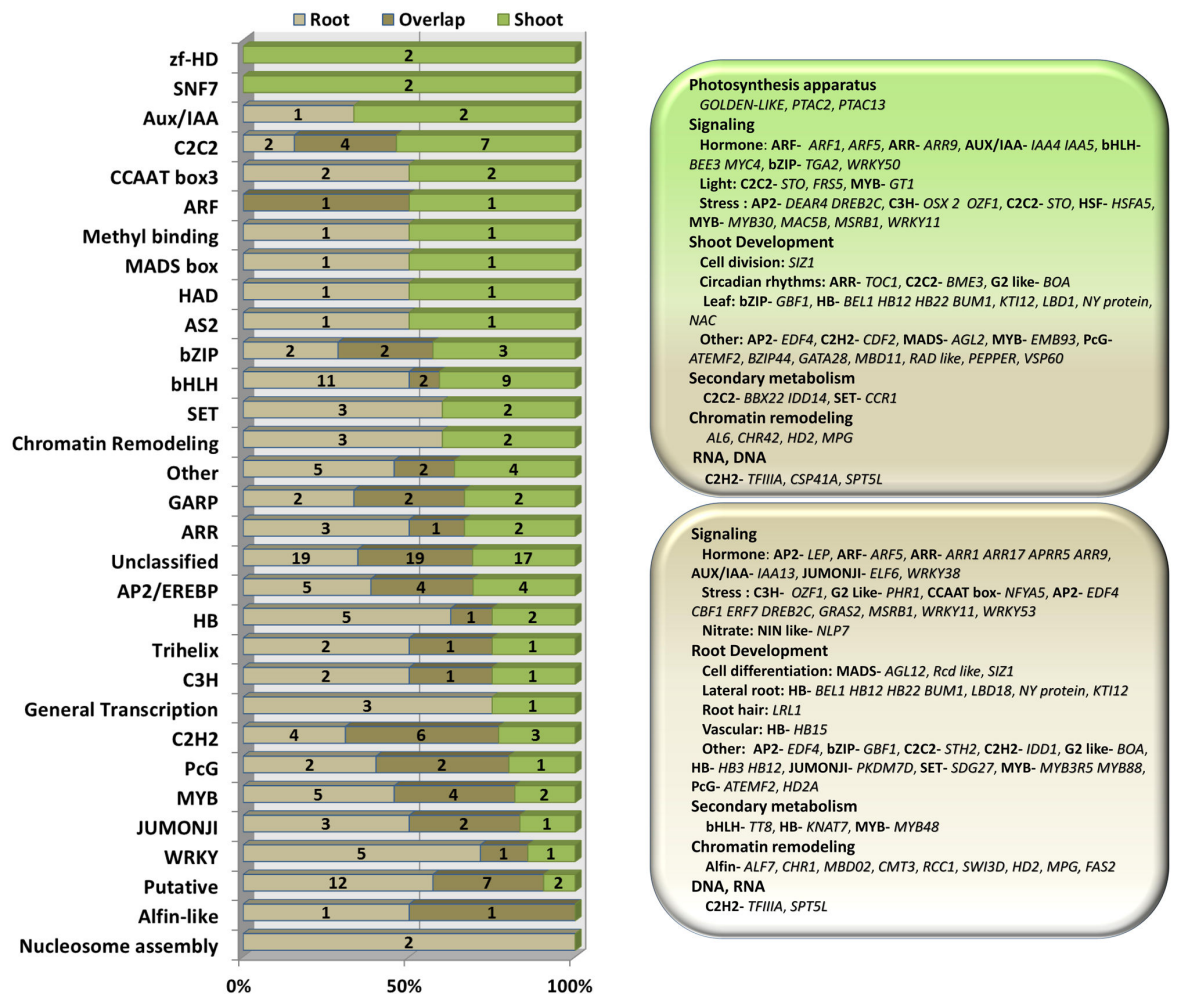
Characterization of the transcription factors of the DEGs in *S. europaea*. (A) Distribution of differentially expressed transcription factor families among *S. europaea* root and shoot. (B) The representative biological functions that the transcription factors may regulate in both root and shoot of *S. europaea* were classified based on previous publications, indicating that the regulation of plant growth and development were active in *S. europaea* under saline conditions.

The sequencing depth of RNA-seq and the high resolution of Tag-sequencing made the characterization of low abundant transcription factor possible to understand the regulatory network of *S. europaea* under saline conditions. Among the DEGs, 171 and 143 transcription factors were found in the roots and in the shoots (Table S12), respectively, and subjected to hierarchical clustering analysis (Figure S9 in File S1). The distribution of transcription factors was consistent with that of DEGs, which reached the peak in both root and shoot at 12 h after NaCl treatment. In general, 215 non-overlapping transcription factors belonged to 31 kinds of families, which participated in the regulation of various physiological process of *S. europaea* under salinity (Figure 7). More than half of the differentially expressed transcription factors, such as cell division, circadian rhythms, lateral root formation, and root hair elongation, were directly or indirectly involved in the growth and development process of *S. europaea* root and shoot, whereas only a small fraction participated in the stress response, which was consistent with our observation that salt treatment could induce the growth of *S. europaea*. We also detected the transcription factors in *S. europaea* shoots that were shown to regulate chloroplast development, light signal transduction, and convergence of plastid signals to the nucleus. 

## Discussion

### Comparative transcriptome analysis of non-model plant could be conducted by next-generation sequencing

Numerous non-model plants that lived in extreme environments have evolved special mechanisms to survive, which have significant scientific research value [51-53]. However, the lack of genome information has significantly restricted further investigation of these plants. The next generation RNA-seq technology has been well developed, which is suitable for large-scale gene expression profiling in non-model organisms.

In this study, RNA-seq was performed to profile the transcriptome of *S. europaea* by using the Illumina Hiseq 2000 platform. A total of 26,266,670 clean reads with the coverage of 2.3 Gb were obtained. A total of 57,151 unigenes were identified after *de novo* assembly, in which 57% was successfully annotated. The C-value of *S. europaea*, which refers to the amount of DNA contained within a haploid nucleus [54], has been determined with the 2C value calculated as 2.75 ± 0.03 pg [55]. Using the formulas to convert the number of nucleotide base pairs to picograms of DNA [56], we predicted the genome size of *S. europaea* as1344.75 ± 14.67 Mb. Considering previous reports of the relationship between genome size and annotated genes [57,58], we found that the assembled annotated unigenes exhibited a comprehensive coverage of the total genes in *S. europaea*. Furthermore, the copy number of tags could be used in the tag-seq method to indicate the expression profiles of the corresponding unigenes; this method can perform better than microarray analysis to quantify gene expression profiles in non-model plants [59,60].

### Key Role of Metabolic Pathways in *S. europaea* Coping with Salinity

GO is an internationally standardized gene functional classification system that provides a standard list to describe the properties of genes in any organism. The GO annotation of *S. europaea* unigenes. We obtained with the help of Blast2GO program [30]. A previous study reported a GO comparison between *Arabidopsis* and its close relative *Thellungiella salsuginea*, a halophyte showing resistance to abiotic stress, to analyze the differences of gene function distribution at a macro level [8]. In the current study, a similar GO comparison was performed between *S. europaea* and *T. salsuginea* to determine the divergence between a euhalophyte that could adapt to saline conditions and a halophyte that showed resistance to such conditions. 

For the GO categories that showed significant difference between species, the terms “response to stimulus” and “biological regulation” were identified to classify more genes of *T. salsuginea* under these two categories. *S. europaea* contained significantly higher amounts of genes in the “metabolic process” and “catalytic activity” categories (Figure S10 in File S1). This divergence of gene distribution at a whole level indicated that *S. europaea*, as a halophyte acclimatize to saline environment, tends to cope with salinity by the metabolic pathways that are often stable and constant rather than the induced response to stress, which is rapid and short lived. 

Numerous genes in *S. europaea* changed their expression profiles after salt treatment. Through gene functional enrichment analysis, these DEGs were screened to determine the important pathways involved. Interestingly, most of the enriched pathways were involved in the primary metabolic pathways that showed consistency with the GO comparison analysis. These pathways could form an interconnected metabolic network that may help reveal the possible mechanism for salt adaptation of this plant. Among the significantly enriched pathways, the remarkable events observed in *S. europaea* are the DEGs involved in cell wall metabolism, chlorophyll and carotenoid biosynthesis, and other plant development and growth-related pathways, which may have special significance for *S. europaea* under saline conditions.

### Salt could affect cell wall metabolism and induce xylem differentiation in *S. europaea* to facilitate sodium assimilation

Plant cell wall is a rigid or sometimes a flexible layer that surrounds several types of cells, providing these cells with structural support and protection. Plant cell wall consists of three layers [61,62]: the middle lamella, which is the outermost layer rich in pectins; the primary cell wall, which is made of cellulose, hemicellulose, pectin, and several proteins for cell wall structure; and the secondary wall, which is a layer inside the primary cell wall that consists of cellulose and lignin. For several parenchymal cells, the primary cell wall is permanent and remains unchanged; for other kinds of cells such as the vascular cells in the xylem, a new layer called the secondary cell wall gradually emerges at the inner side of the primary cell wall [61,62].

**Figure 8:**
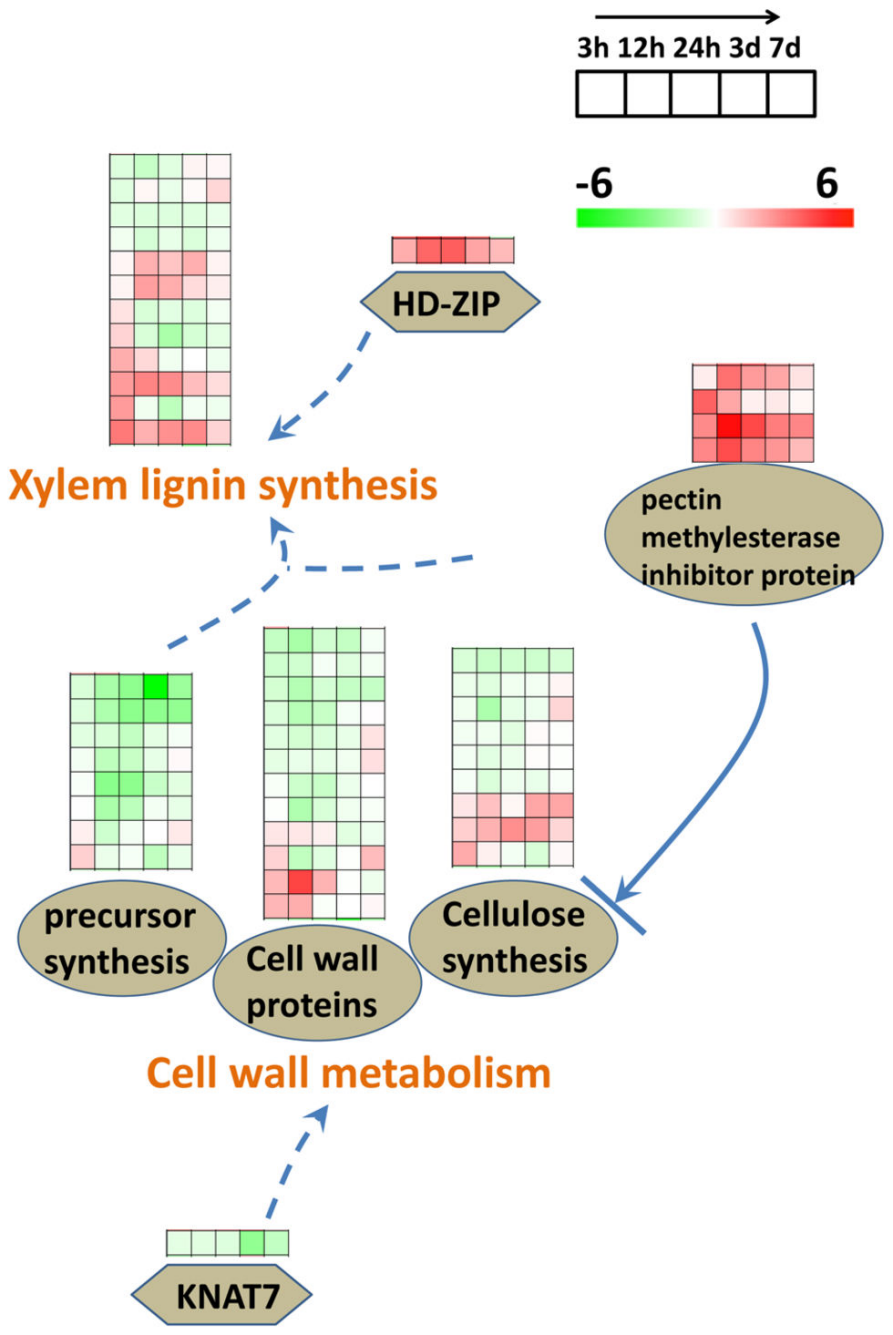
Pathways of cell wall metabolism were revealed by gene functional enrichment analysis. The log_2_ transformed ratio was used to represent the fold change for the expression of different genes. A significant majority of the genes encoding cell wall precursor proteins and cell wall structure proteins as well as the biosynthesis of cellulose were downregulated after salt treatment. The genes of pectin methylesterases inhibitor family proteins showed upregulation under saline conditions, which decreased the level of methyl esterification of pectin, thus affecting the cellulose biosynthesis. A root transcription factor KNAT7 was reduced under saline conditions and negatively regulated the synthesis of primary cell wall. Many of the genes involved in the lignin biosynthesis were up-regulated in *S. europaea* under saline conditions, which was consistent with previous reports that reduced cellulose synthesis, possibly induces plant lignification. The gene expression of a Homeobox family protein, HD-ZIP, increased in abundance after salt treatment, which can induce xylem development in vascular bundle as reported.

For the root of *S. europaea* under saline conditions (Figure 8), the genes involved in cell wall precursor synthesis, such as the UDP-L-rhamnose synthase, were downregulated, thereby producing rhamnose, the precursor of pectin [63]. In addition, a large majority of the genes encoding cell wall structure proteins of the primary cell wall and the cellulose synthesis proteins were down-regulated after salt treatment. The genes of pectin methylesterases inhibitor family proteins were up-regulated under saline conditions, which decreased the level of methyl esterification of pectin and affected the normal function of pectin by inhibiting pectin methylesterase activity [64-66]. From the gene expression analysis for cell wall metabolism genes, we can deduce that the biosynthesis of the middle lamella and the primary cell wall was repressed, so does the biosynthesis of cellulose, the secondary cell wall key component. Previous reports have proven that reduced cellulose synthesis possibly induces plant lignification [67,68], which is consistent with our observation that numerous genes involved in lignin biosynthesis were up-regulated in *S. europaea* under salinity. Furthermore, we found that several transcription factors were differentially expressed to regulate the process of cell wall metabolism. For example, the expression of a root transcription factor KNAT7 was reduced under saline conditions, which negatively regulated the synthesis of primary cell wall [69,70]. The gene expression of a Homeobox family protein HD-ZIP significantly increased after salt treatment, which can induce the development of xylem in vascular bundle as reported [71]. The differentially expressed genes involved in cell wall metabolism agreed well with the vessel differentiation increment in xylem, as observed in the transversal section of *S. europaea* shoot. The lignified vessel in xylem is the main passage for water and mineral elements assimilation. Therefore, the accumulation of large amount of salt in *S. europaea* shoot under salinity calls for a powerful transport system from root to shoot, which may be one of the most important strategies of this halophyte adapting to salinity.

Sodium is an element toxic to plants under saline conditions and can induce osmotic stress and affect enzymatic activity that harm plant cells [72-74]. For glycophytes, one of the strategies for plants to cope with salinity is to prevent Na^+^ or Cl^−^ from entering the cell membrane [73]. However, for the euhalophyte, sodium is an indispensable element that always accumulated to the aerial part of plant [12,14,75]. Therefore, time serial analysis on sodium assimilation and distribution patterns would help in understanding how halophyte copes with salinity at the initial stage after treatment.

At 3 h after NaCl treatment, the total sodium element content started to rise in the root, which is accompanied by the significantly increased number of DEGs. The total sodium element content of the shoot remained unchanged. However, the sodium content was already observed increasing in the shoot xylem. This observation means that sodium had not been accumulated to shoot yet, although it was started to be transported from root to shoot through the xylem at 3 h of the NaCl treatment. The distribution of DEGs was more likely to be connected with the total sodium content in both root and shoot. The number of DEGs in the root at 3 h after treatment was significantly larger than it was in the shoot. This result means that, as the first site facing salinity, the root could respond to salt signal earlier than the shoot. At 12 h after NaCl treatment, the sodium content significantly increased in the shoot, which was accumulated in the parenchymal cell of the exodermis. The number of shoot DEGs at this point showed a significant increase compared with that at 3 h after treatment, which was also the peak number throughout the whole treatment period for both shoot and root. This case indicates that 12 h after treatment was the key stage for salt response in *S. europaea*, which was also accompanied with the start of sodium accumulation in the shoot parenchyma. 

### Salt inhibits chlorophyll biosynthesis while it activates the expression of genes involved in electron transfer in chloroplast

After the results of gene functional enrichment analysis of DEGs were screened, we determined that the pathways of tetrapyrrole and isoprenoid biosynthesis were significantly enriched in the shoot of *S. europaea*, which were also the main enriched terms for the shoot k3 clusters, showing down-regulated gene expression pattern after salt treatment.

**Figure 9:**
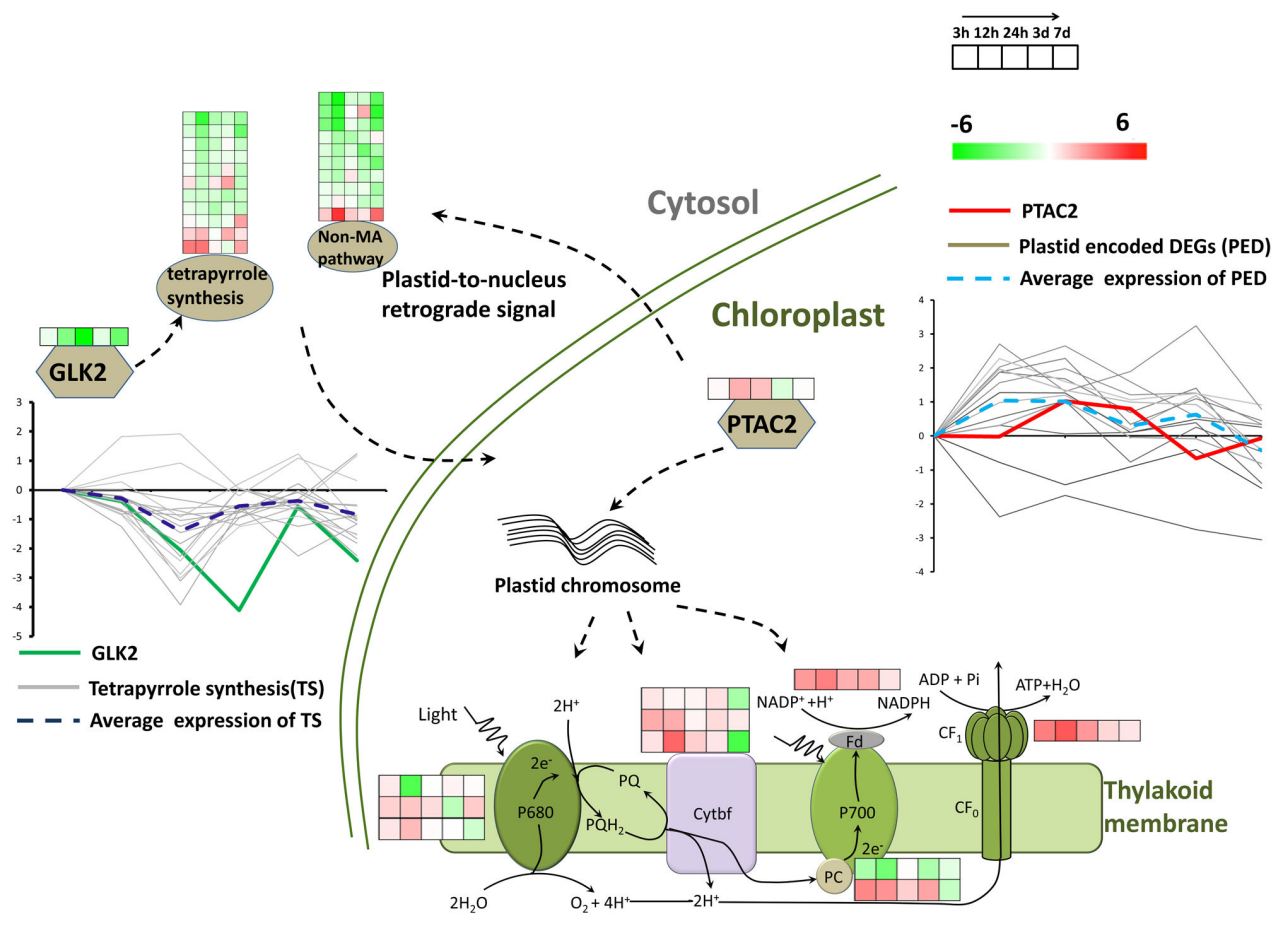
Possible model of the regulated pattern between GLK2 and pTAC2. The log_2_ transformed ratio was used to represent the fold change of the expression of different genes. Many of the genes in the non-mevalonate and tetrapyrrole biosynthesis pathways, which produce photosynthetic pigments in the chloroplast, showed reduced expression pattern after salt treatment, as depicted in the graph, with the average expression pattern highlighted by the blue dash line. A plastid-located transcription factor GLK2 was observed to have very similar gene expression patterns (green solid line) with the chlorophyll biosynthesis genes. Inside the chloroplast, many of the chloroplast chromosome encoded genes showed up-regulated expression patterns under salinity, with the average expression pattern highlighted by the light blue dash line. The transcription factor pTAC showed similar expression patterns (red solid line) as the photosystem proteins. The crosstalk may exist between the transcription factor pTAC and GLK.

The non-mevalonate pathway, which belongs to the isoprenoid synthesis pathway, participates in carotenoid biosynthesis in chloroplast [76,77]. Tetrapyrrole biosynthesis also takes place in the chloroplast, which is the key pathway for producing chlorophyll [78]. The reduced expression of the genes in these pathways indicated that the photosynthetic pigments were inhibited at the early stage of salt treatment for *S. europaea*. However, the PSII activity measured by characterizing the chlorophyll fluorescence was unchanged. This result means that although the pigment synthesis was inhibited, the electron transfer it mediated was unaffected. A previously reported transcription factor GLK2, which can accept the signal from plastid to regulate the gene expression of the nuclear-encoded plastid-located proteins [79,80], exhibited similar downregulated gene expression patterns to the genes related to chlorophyll biosynthesis. Another study has revealed that GLK2 can determine chlorophyll accumulation and distribution in the developing fruit of tomato [81]. This result is consistent with our results, in which *GLK2* was co-regulated with the genes related to chlorophyll biosynthesis. After a prolonged salt treatment, the shoot color of *S. europaea* possibly became light green [23], which resembled the fruit light green phenotype of the tomato GLK2 mutant. This result indicated that salt may function as an environmental inhibitor for the expression of *GLK2*, which rigorously controlled the biosynthesis of chlorophyll in *S. europaea* under salinity (Figure 9). 

The gene expression of photosystem proteins in *S. europaea* was further analyzed. The chloroplast chromosome encoded genes (Table S13), such as several PS I and PS II pigment binding proteins, the b_6_f complex proteins, and ATPase synthase CF1 subunit, showed significantly induced expression. We determined that a transcription factor pTAC, which was reported in transcriptionally active plastid chromosome required for plastid gene expression [82,83], showed up-regulated expression patterns as the photosystem proteins displayed. Figure 9 shows the comparison between their similar co-regulated expression patterns, indicating that pTAC may activate the genes expression of photosystem proteins in *S. europaea* under saline conditions.

The crosstalk between pTAC and GLK has not been reported yet. Our gene expression analysis results indicated a proposed model that salt treatment could induce the expression of *pTAC*, which activated the expression of photosystem genes encoded by the chloroplast chromosome. The signal may relay outside the chloroplast, which suppressed *GLK* expression, and finally reduced the gene expression of chlorophyll biosynthesis enzymes. This case means that life will always maintain an energy-efficient state. If the ability to capture light energy for *S. europaea* under salinity could be maintained by the induced expression of plastid chromosome encoded photosystem genes, which will not require too much pigment for binding, then it will inevitably reduce chlorophyll biosynthesis and terminate the energy-consuming state. 

### Other important enriched pathways in *S. europaea*

Other important pathways involved in energy metabolism were also enriched in *S. europaea* (Figure S13 in File S1). We analyzed the energy metabolism genes and found that most key enzymes in the Calvin cycle, such as Rubisco, GAP, ALD, FBP, and REB, showed a significant decrease in gene expression after 12 h of salt treatment and then gradually recovered. However, carbon assimilation efficiency was observed and not altered by measuring the gas exchange parameters. The downregulation of these genes at 12 h was likely due to the dehydration stress induced by the elevated sodium content in the root at the early stage of salt treatment, which may push up the root osmotic potential. This result was consistent with the study of a salt-resistant *Populus* species, which also showed a decreased expression of metabolism genes in the chloroplast within 12 h of salt treatment [84]. We observed the downregulation of starch synthesis genes accompanied with the upregulation of starch degradation genes. This observation indicated the possibility of starch breakdown, which was proven by measuring the starch content at different time intervals (Figure S11 in File S1) based on the reported protocols [85]. This case indicated that the energy metabolism in *S. europaea* shoot is under an active state. The glucose produced in photosynthesis, which was not turned to starch, together with the glucose derived from amylolysis, was transported out of the chloroplast for the energy consumption needed by the growth and development of *S. europaea* under saline conditions. Further analysis revealed the down-regulated expression of several genes involved in glycolysis at the early stage of salt treatment, which is the major glucose catabolism pathway. We observed that the up-regulated gene expression of the rate-controlling enzymes participated in the pentose phosphate pathway, which is an important alternative to glycolysis, producing not only the reducing power but the intermediate carbon skeletons for various biochemical reactions, such as ribose-5-phosphate (R5P) that is used in the synthesis of nucleotides and nucleic acids [86]. This glucose metabolism pathway modulation enables *S. europaea* to manipulate better the numerous biochemical changes, which reflects the subtle regulatory mechanism of metabolism event at the initial stage after salt treatment.

Other genes were also involved in signaling and plant hormone metabolism, playing key roles in *S. europaea* response to salinity (Figures S12 and S13 in File S1). A succession of signaling pathways were triggered in *S. europaea* at the initial stage after salt treatment. Among these pathways, the calcium-signaling pathway, which transmits its signal through two kinds of binding proteins, calcium-binding proteins, and calmodulin-binding proteins [87], were differentially expressed and enriched, especially the up-regulation of gene expression patterns of the calcium binding proteins under salinity, which were reported to regulate the activities of membrane channels [88,89]. In our experiment, numerous ion channels in plasma membranes and vacuole showed differently expressed patterns after treatment. For example, CLC, an anion channel that can transport chloride and nitrate ions in plant [90,91], showed induced gene expression pattern after salt treatment. This result indicated that CLC is a kind of transporter that can be activated in both root and shoot under saline conditions [90,91].

For the differentially expressed hormone metabolism genes, the ethylene biosynthesis genes and ethylene response transcription factors were significantly enriched in the root, with the gene expression level largely induced. As a plant hormone, ethylene cannot only regulate the lateral root development with auxin but can also participate in stress response signal transduction [92-94]. Further analysis for the expression of ethylene-responsive element genes indicated that nine AP2 transcription factors were up-regulated at the early stage of salt treatment, while recovered at 7 days of treatment. Most of these AP2 proteins were reported to be involved in osmotic dehydration stress [95,96], which indicated that ethylene-signaling pathway may play an important role in the root to relieve the osmotic stress caused by an increased soil osmotic potential at the initial stage of salt treatment. Furthermore, DEGs involved in auxin-signaling pathway took a more important part in the response of *S. europaea* shoot under saline conditions; the majority of these DEGs showed increased expression patterns. Auxin signal has an important function in coordinating numerous processes in plant development and growth from seed germination to flowering and fruit ripening process [97-99]. The results indicated that the gene expressions of auxin responsive transcription factors (ARF) and auxin induced proteins Aux/IAA family were significantly induced after salt treatment in *S. europaea* shoot. Through further analysis of the auxin-related transcription factors, we determined that three NAC family proteins, which function closely interacted with auxin to regulate plant development [100-102], were also induced in gene expression of *S. europaea* shoot at different periods after treatment. In addition, numerous plant growth and development-related transcription factors exhibiting induced gene expressions at different extents in different stages after salt treatment were noted. This condition indicated that salt could promote auxin signaling pathways, which were maintained at different stages after salt treatment, and interacted with different transcription factors to promote the development of *S. europaea*.

## Supporting Information

File S1 **This file contains Figure S1, Figure S2, Figure S3, Figure S4, Figure S5, Figure S6, Figure S7, Figure S8, Figure S9, Figure S10, Figure S11, Figure S12, and Figure S13.**
(DOCX)

Table S1 **Unigene annotation compared with non-redundant protein database at NCBI.**
(XLS)

Table S2 **Unigene annotation compared with Swissprot protein database.**
(XLS)

Table S3 **Unigene annotation compared with COG protein database at NCBI.**
(XLS)

Table S4 **Unigene annotation compared with KEGG database.**
(XLS)

Table S5 **Unigene GO annotation by the Blast2GO program.**
(XLS)

Table S6 **Unigene annotation based on the MapMan classification.**
(XLS)

Table S7 **Differentially expressed unigenes in the root samples after NaCl treatment at different time intervals.** “FDR ≤ 0.001 and the absolute value of log2Ratio ≥ 1” was set as the threshold for the library of tags to determine the significance of gene expression.
(XLS)

Table S8 **Differentially expressed unigenes in the shoot samples after NaCl treatment at different time intervals.** “FDR ≤ 0.001 and the absolute value of log2Ratio ≥ 1” was set as the threshold for the library of tags to determine gene expression significance.
(XLS)

Table S9 **Primers used in qRT-PCR and RNA *in situ* hybridization.**
(XLS)

Table S10 **k-means clustering of the DEGs in *S. europaea* roots treated with NaCl at different time intervals.** The DEGs and their MapMan annotations in the six clusters were respectively shown.
(XLS)

Table S11 **k-means clustering of the DEGs in *S. europaea* shoot treated for different time intervals with NaCl.** The DEGs and their MapMan annotations in the six clusters were respectively shown.
(XLS)

Table S12 **Differentially expressed transcription factors in *S. europaea* root and shoot after salt treatment.** Among the DEGs, 171 transcription factors were found in the root and 143 were found in the shoot.
(XLS)

Table S13 **Differentially expressed chloroplast chromosome encoded genes in *S. europaea* shoots annotated by the MapMan classification.**
(XLS)
