# Supplementary material for: Transcriptome Analysis of Salicornia europaea under Saline Conditions Revealed the Adaptive Primary Metabolic Pathways as Early Events to Facilitate Salt Adaptation
Source: PLoS One. 2013 Nov 12;8(11):e80595. doi: 10.1371/journal.pone.0080595 (PMC3827210; doi:10.1371/journal.pone.0080595)

**Figure S1.** Design of NaCl treatment of *S. europaea*. The plants were treated at different time intervals and then harvested at the same time to allow the plants have the same biological age and growth rhythm after treatment.


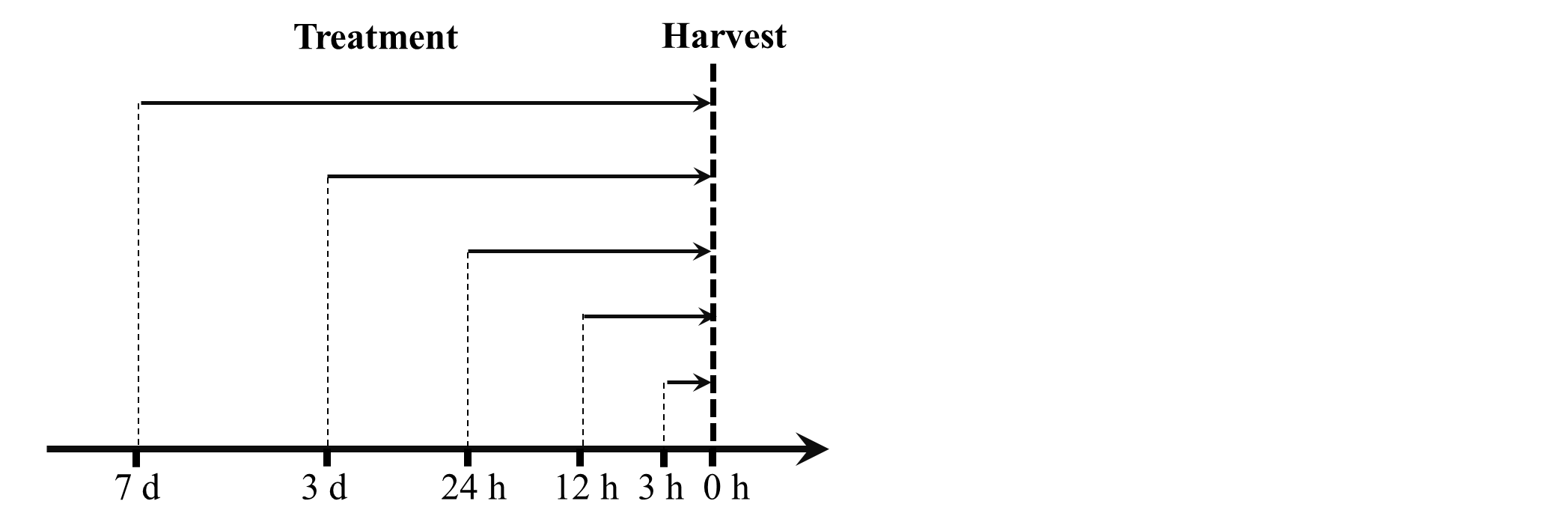


**Figure S2.** Functional annotation of *S. europaea* unigene by (A) GO classification, (B) COG function classification, and (C) MapMan classification.


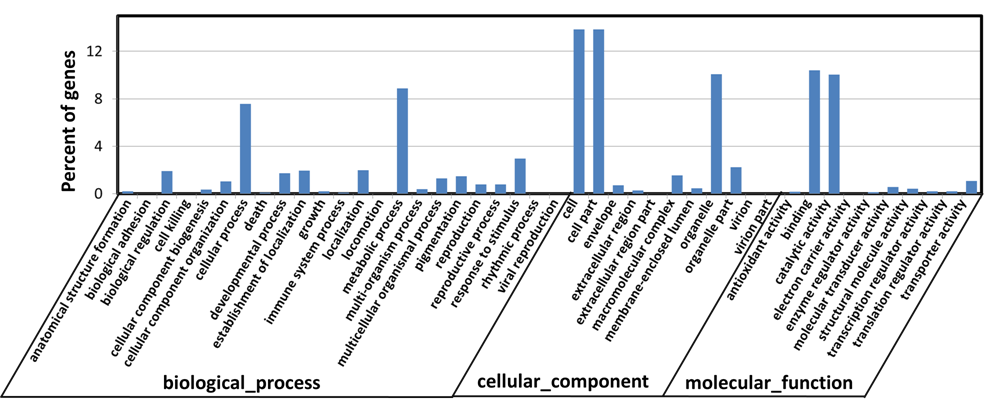


**A**


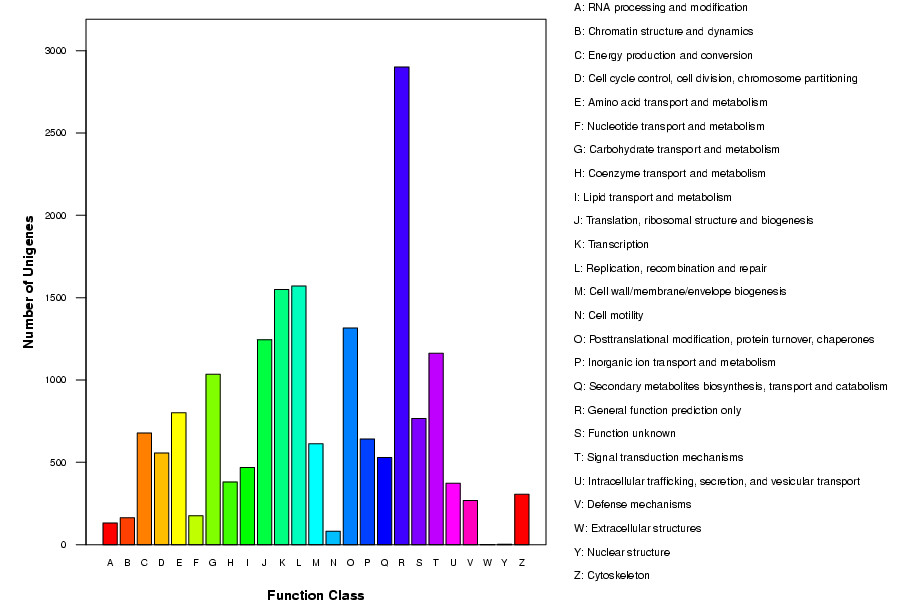


**B**


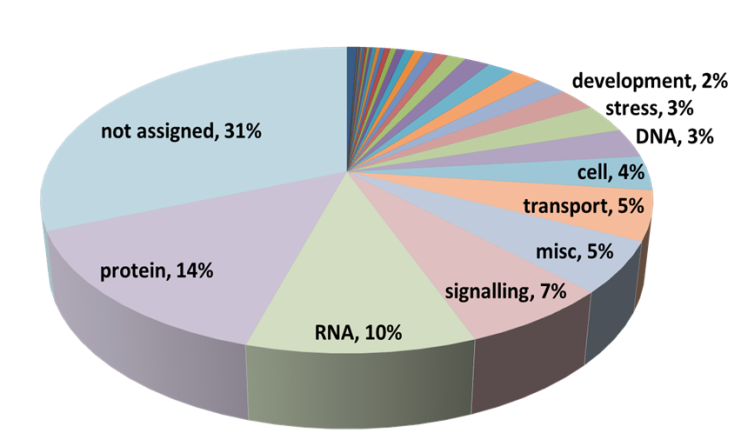


**C**

**Figure S3.** Quality evaluation of digital gene expression sequencing. (A, B) Sequencing saturation analysis of root and shoot samples; (C, D) Distribution of total tags in root and shoot samples; and (E, F) Distribution of distinct tags in root and shoot samples.


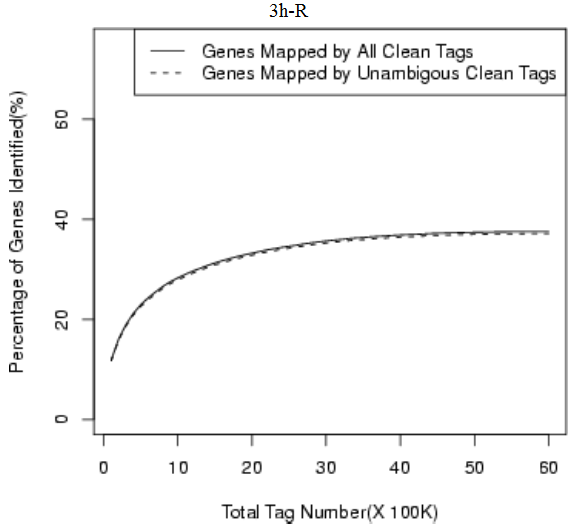

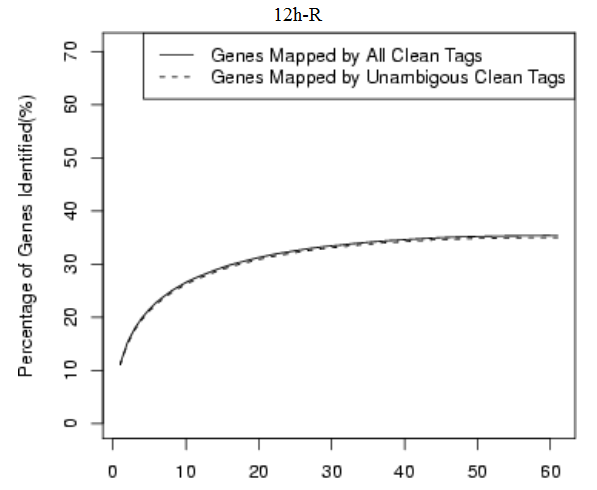

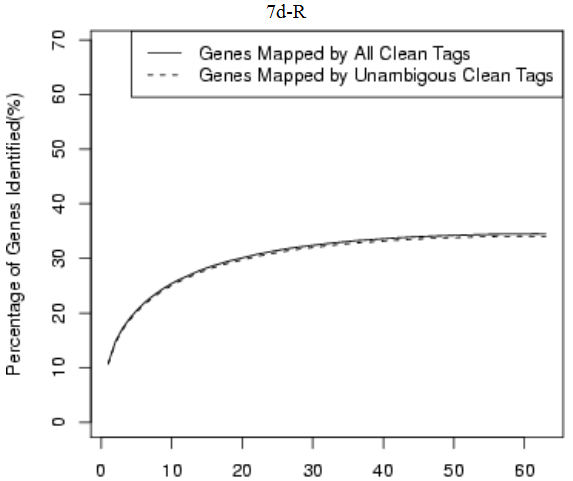

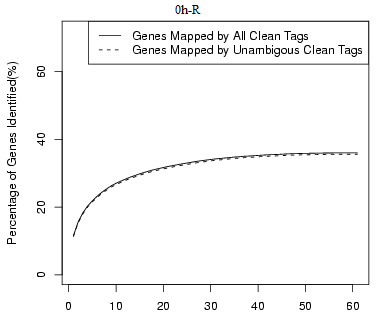


**A**


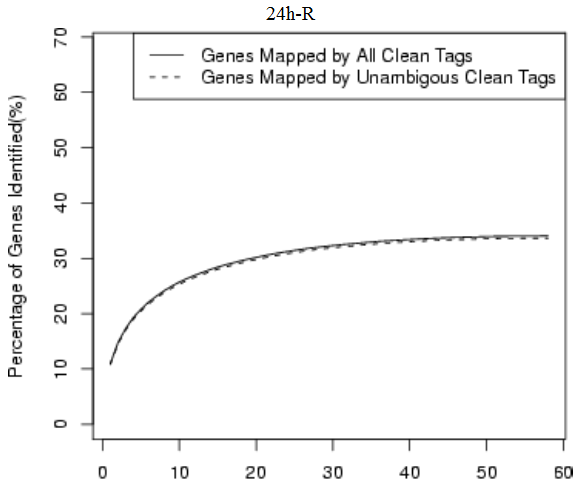

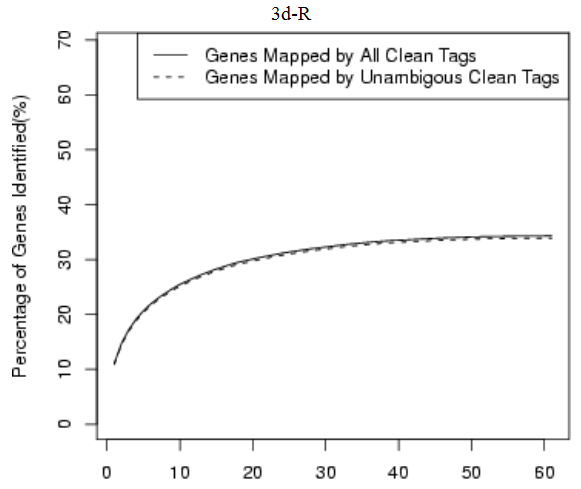


**A**


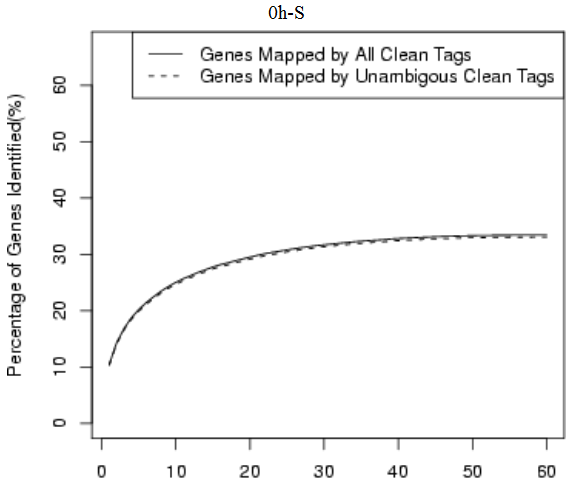

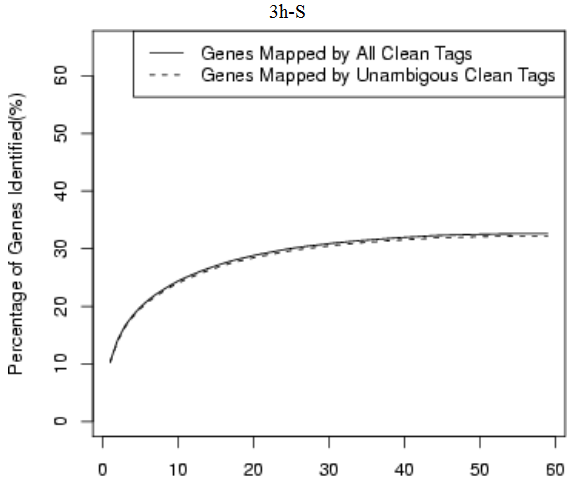

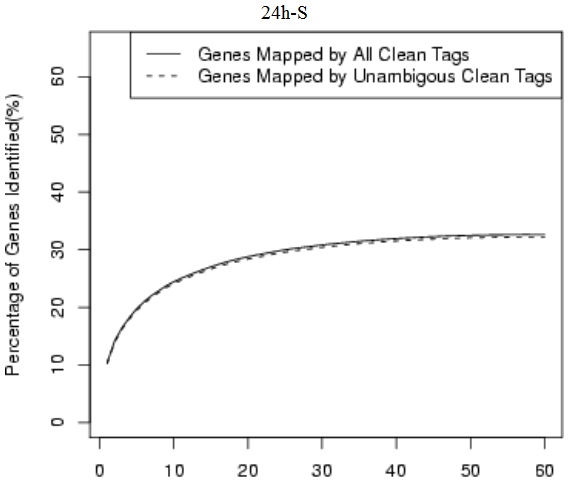

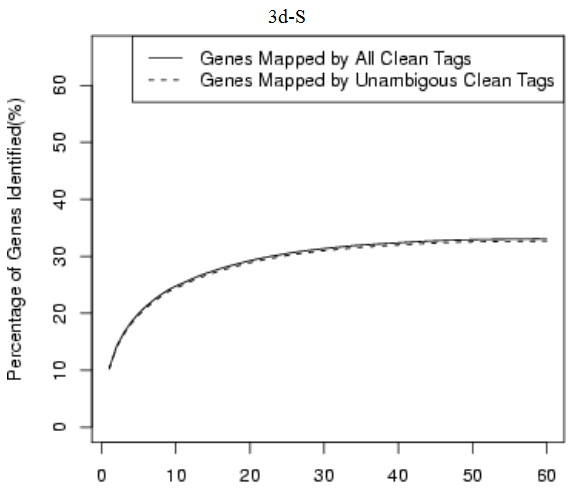

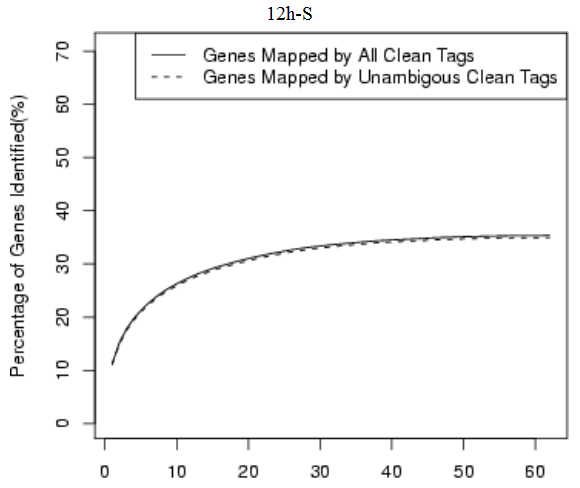

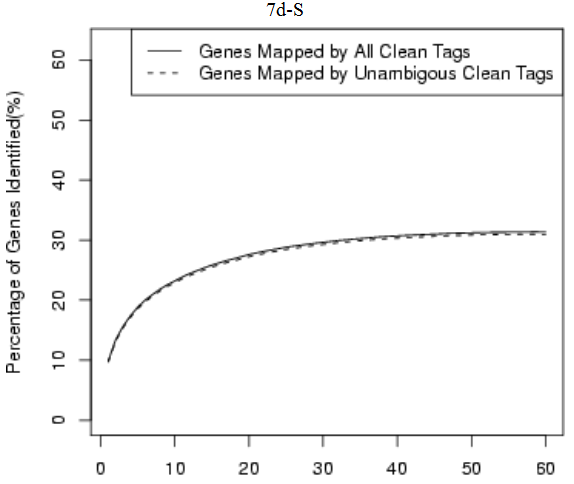


**B**


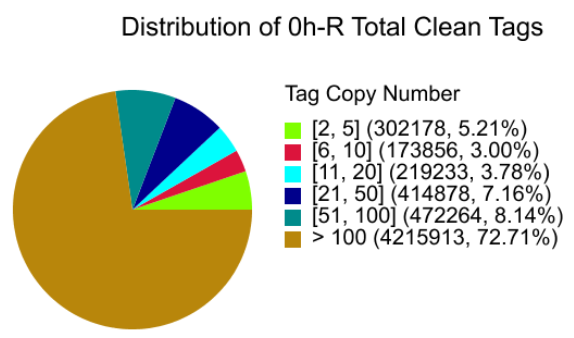

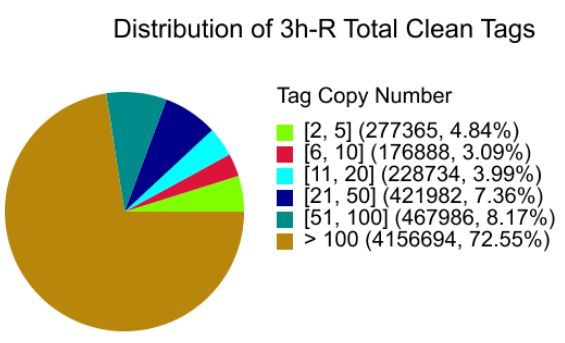

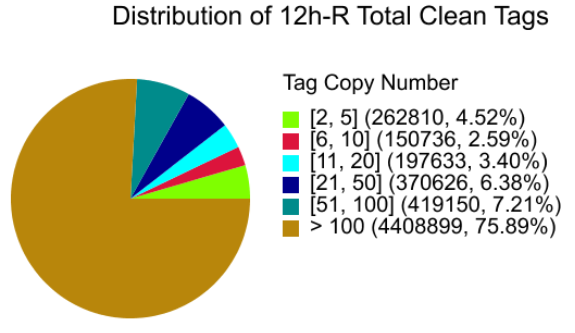

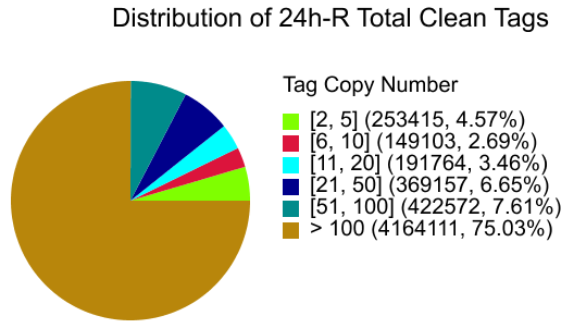

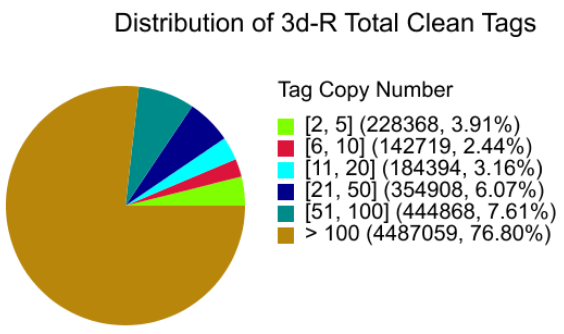

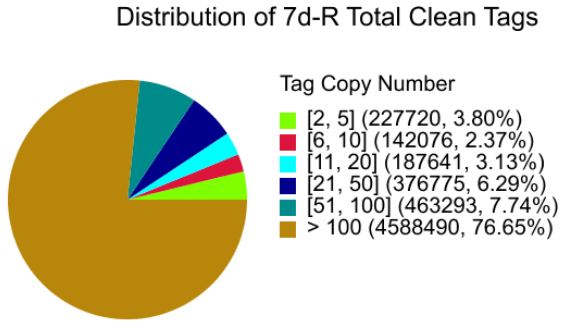


**C**


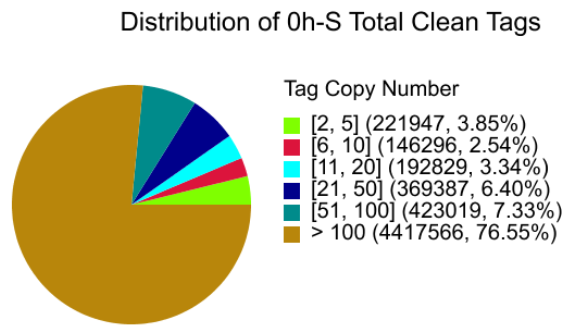

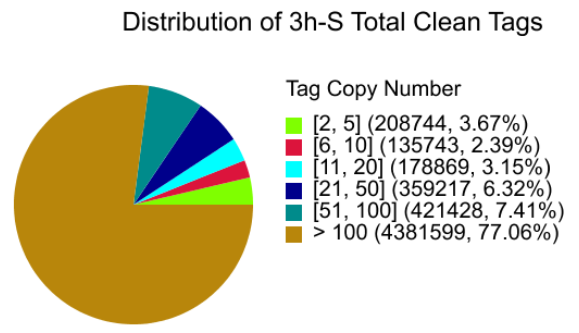

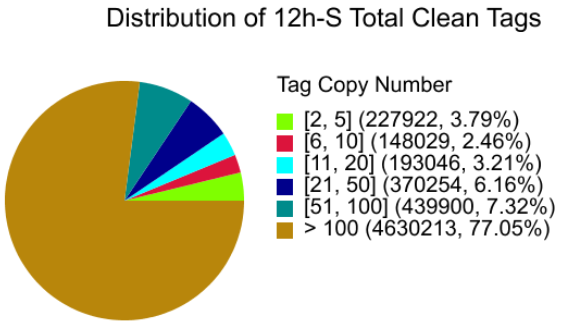

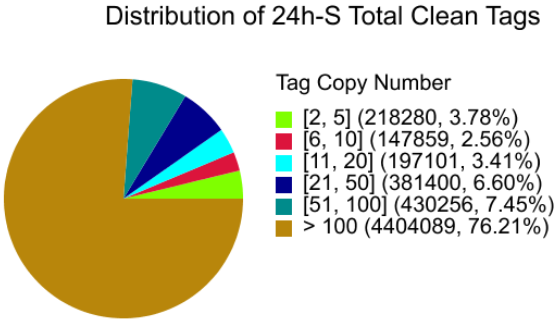

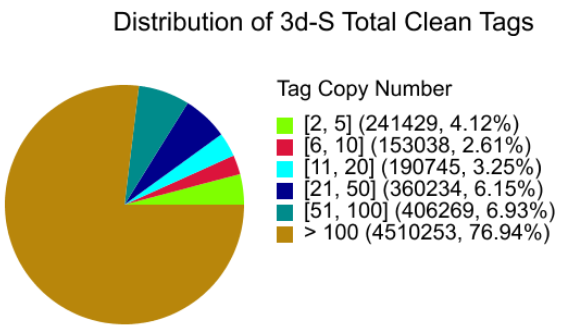

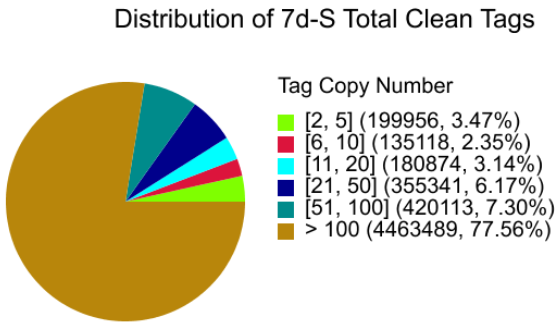


**D**


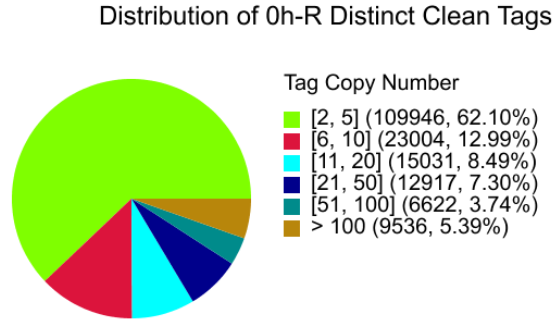

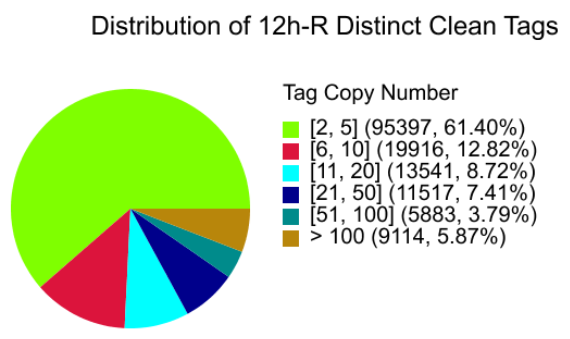

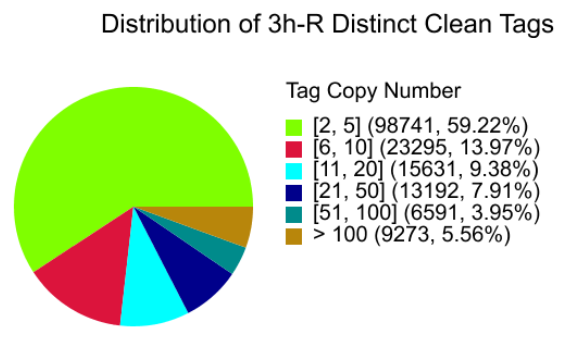

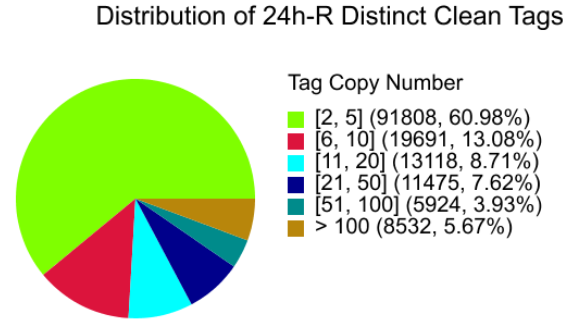

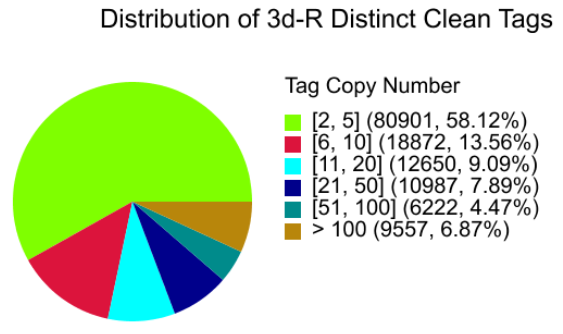

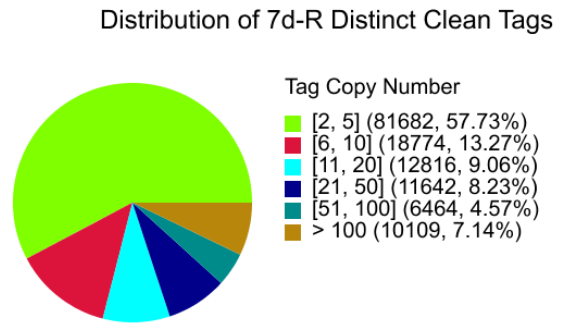


**E**


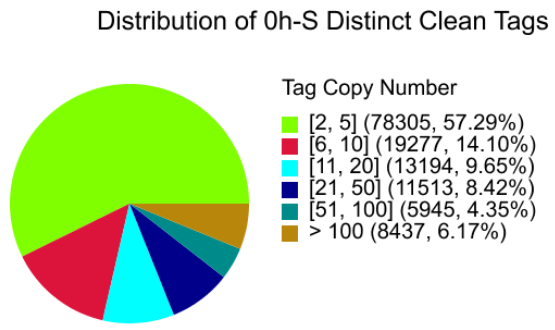

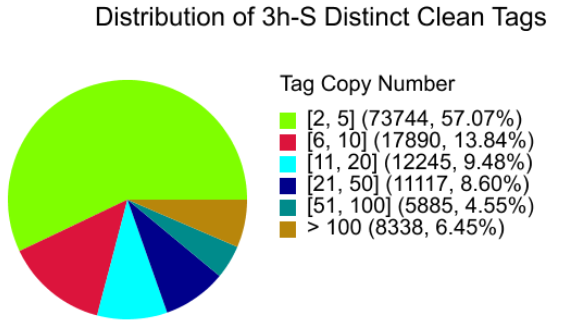

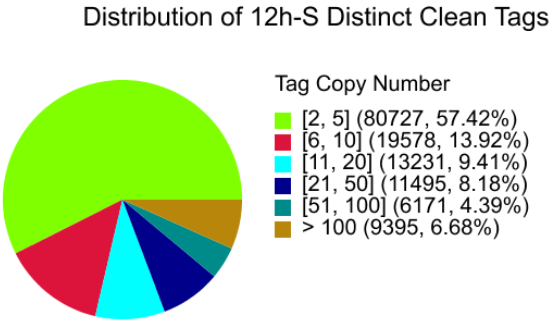

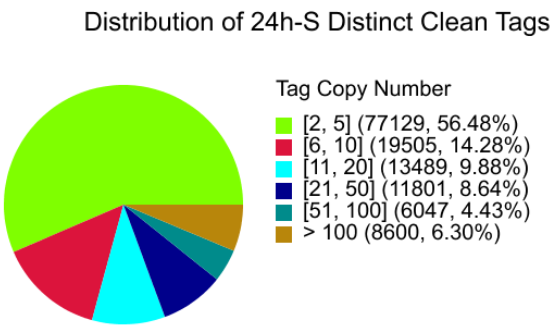

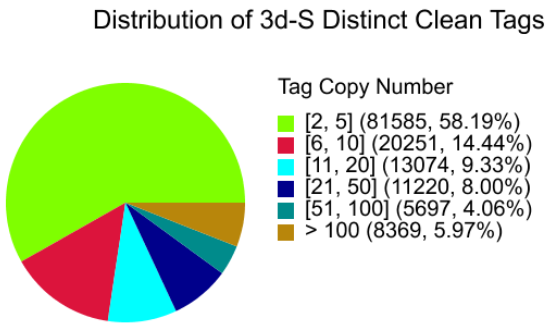

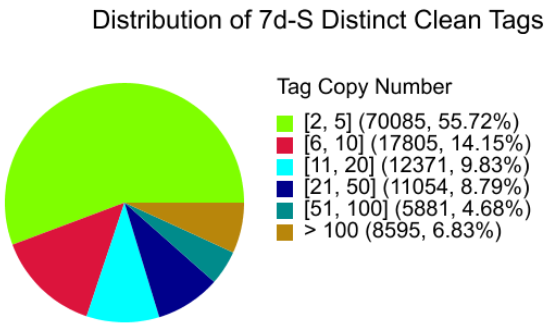


**F**

**Figure S4.** Verification of digital gene expression analysis by qRT-PCR. The differentially gene expressions of six randomly selected unigenes from the root and the shoot were verified by qRT-PCR and the correlation coefficients were calculated.


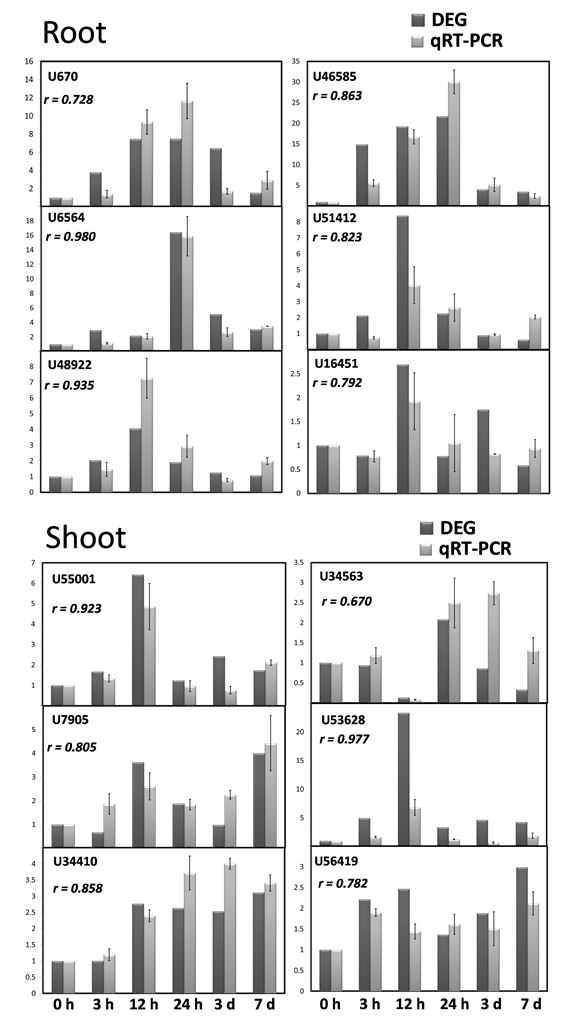


**Figure S5.** Verification of expression distribution of unigenes by RNA *in situ* hybridization analysis. The gene-expression distributions at the tissue level of two unigenes, an auxin transporter PIN8 (A) and a proton-dependent oligopeptide transporter (B), were verified by RNA *in situ* analysis in the root and the shoot, respectively.


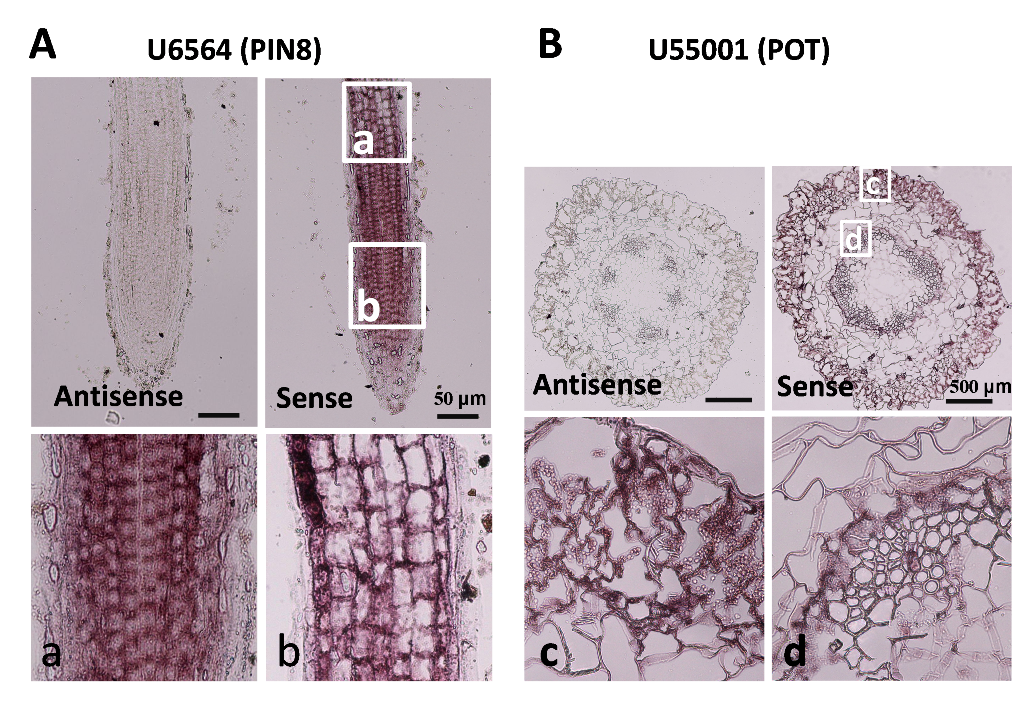


**Figure S6.** Gene-significant enrichment analysis of *S. europaea* root at different time intervals of NaCl treatment based on the second level of MapMan classification. Red and green boxes represent upregulated and downregulated genes, respectively.


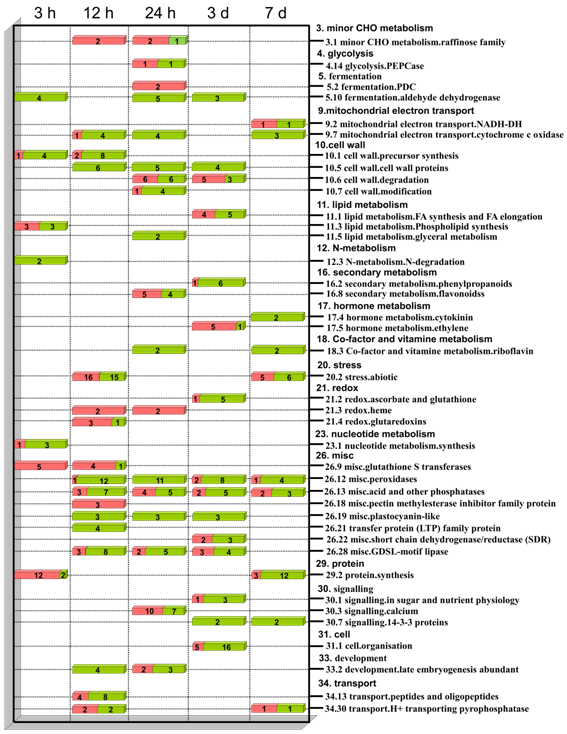


**Figure S7.** Gene-significant enrichment analysis of *S. europaea* shoot at different time intervals of NaCl treatment based on the second level of MapMan classification. Red and green boxes represent upregulated and downregulated, respectively.


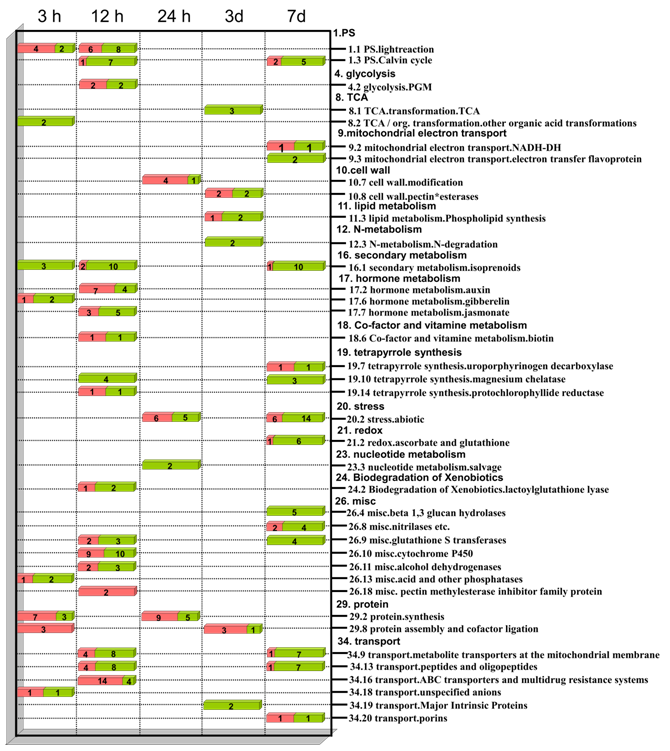


**Figure S8.** FOM of different k-means cluster numbers for all DEGs in the shoot (A) and the root (B) at different time intervals of NaCl treatment.

**FOM value *vs* No. of clusters**


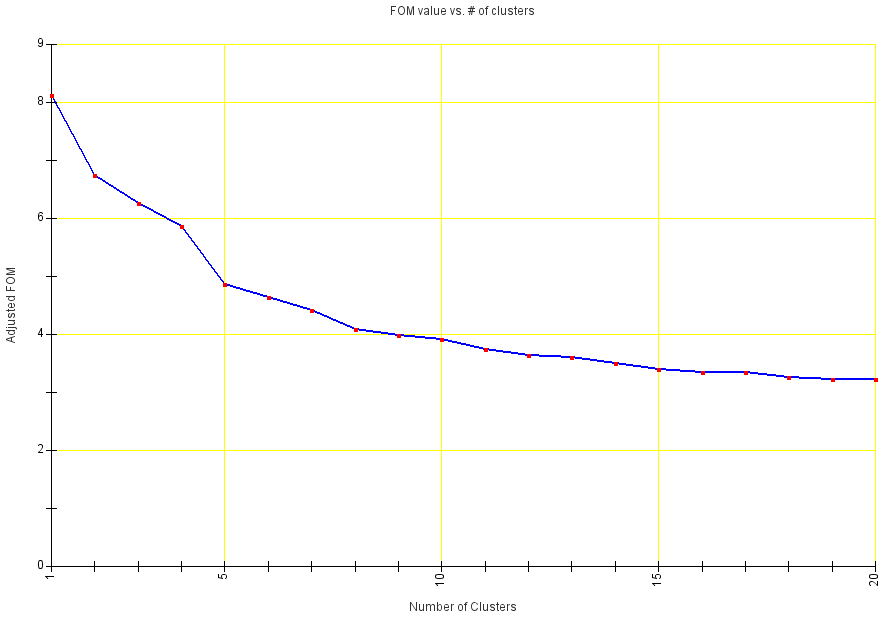


**Adjusted FOM**


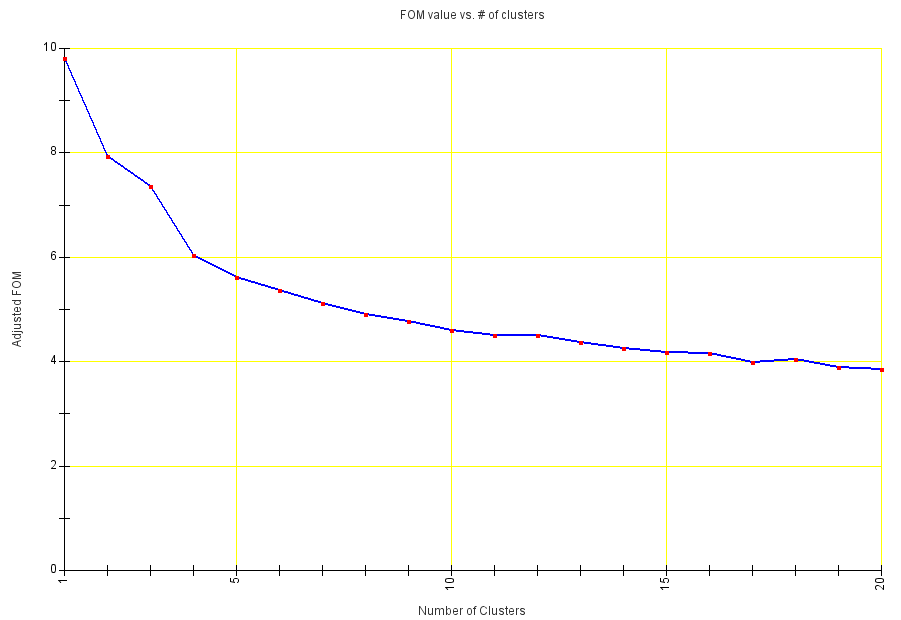


**Adjusted FOM**

**Number of clusters**

**Figure S9.** Hierarchical clustering analysis of differentially expressed transcription factors in *S. europaea* root and shoot at different time intervals of NaCl treatment.


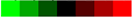


**-6 -3 -1 0 1 3 6**


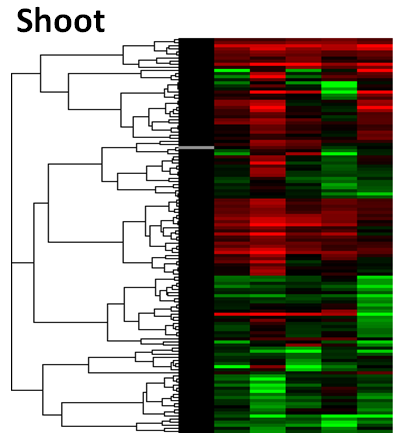


**0h 3h 12h 24h 3d 7d**


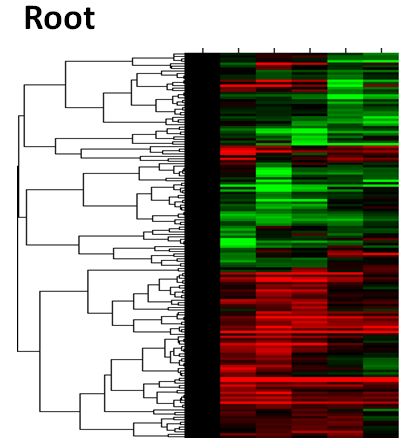


**0h 3h 12h 24h 3d 7d**

**Figure S10.** GO comparison of *S. europaea* and *T. salsuginea*. Blast2GO results of *S. europaea* unigenes were mapped to categories in the second level of GO terms and then compared with the GO terms of *T. salsuginea*. Fisher’s exact test was used to assess the significance of differences for the distribution of gene numbers in different GO categories of the two species. Those with P values below 0.01 were considered as having significant differences. Red “*” indicates that *T. salsuginea* has significant genes in this GO category compared with *S. europaea*; blue “*” shows opposite results.


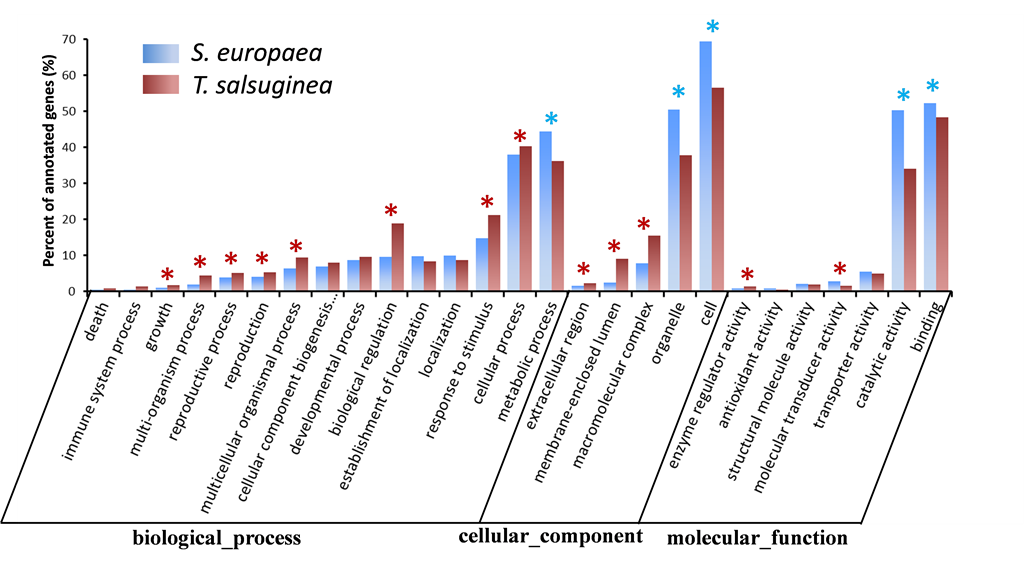


**Figure S11.** Starch content measurement in the shoot of *S. europaea* treated with NaCl for different time intervals. Data are presented as mean ± SD (*n* = 4).


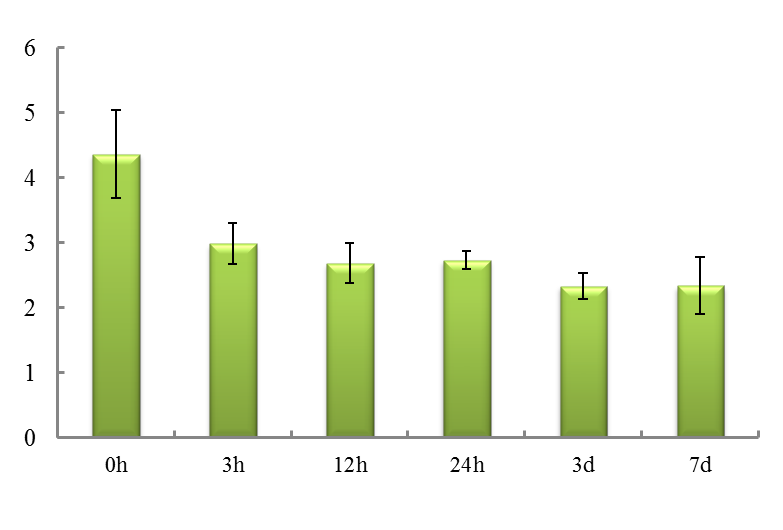


**mg / g FW**

**Figure S12.** Network of important physiological and biochemical changes in *S. europaea* root revealed by gene functional enrichment analysis.


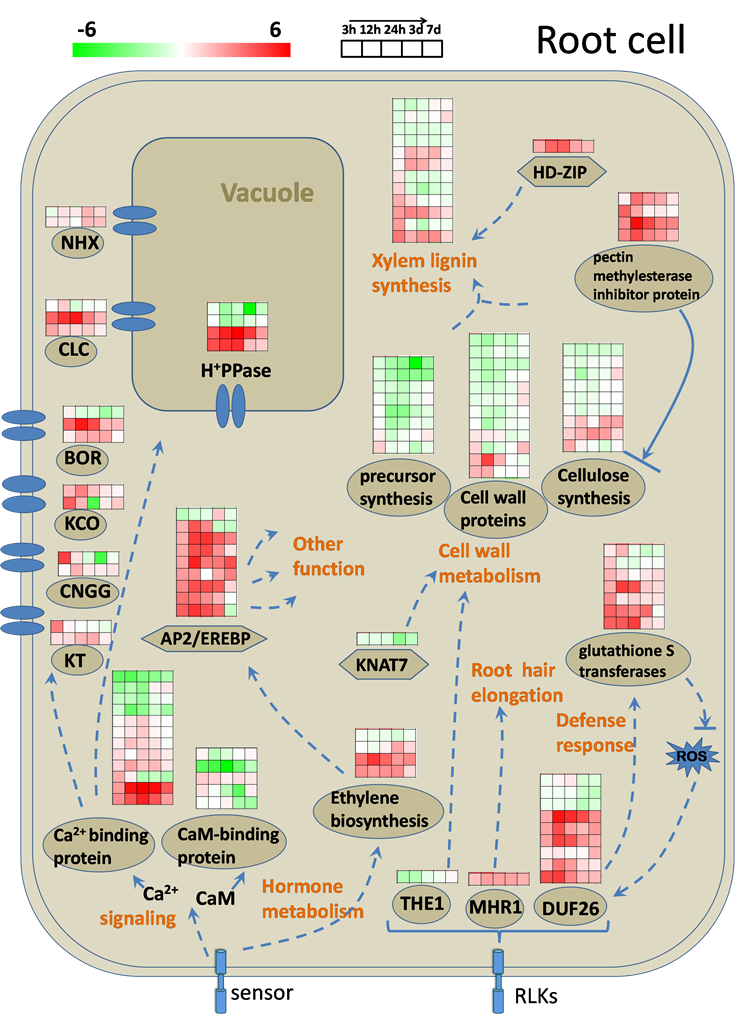


**Figure S13.** Network of important physiological and biochemical changes in *S. europaea* root revealed by gene functional enrichment analysis.


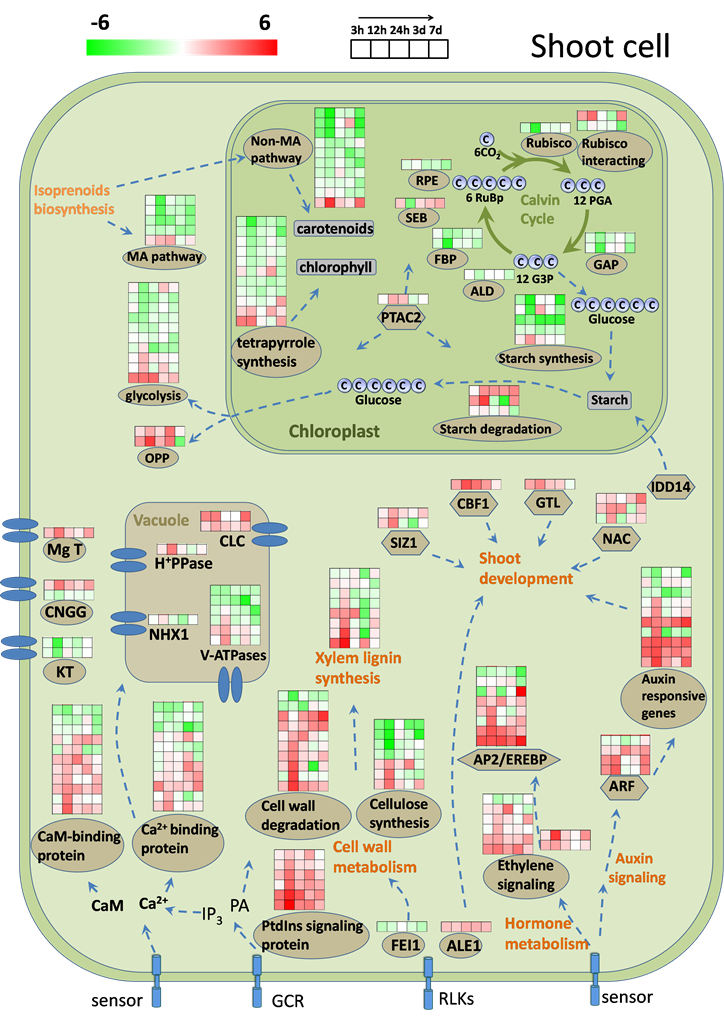

Supplement: File S1 — This file contains Figure S1, Figure S2, Figure S3, Figure S4, Figure S5, Figure S6, Figure S7, Figure S8, Figure S9, Figure S10, Figure S11, Figure S12, and Figure S13. (DOCX) [file pone.0080595.s001.docx]
